# A novel tumor mutational burden estimation model as a predictive and prognostic biomarker in NSCLC patients

**DOI:** 10.1186/s12916-020-01694-8

**Published:** 2020-08-26

**Authors:** Yanhua Tian, Jiachen Xu, Qian Chu, Jianchun Duan, Jianjun Zhang, Hua Bai, Zhenlin Yang, Wenfeng Fang, Liangliang Cai, Rui Wan, Kailun Fei, Jie He, Shugeng Gao, Li Zhang, Zhijie Wang, Jie Wang

**Affiliations:** 1 State Key Laboratory of Molecular Oncology, Department of Medical Oncology, National Cancer Center/National Clinical Research Center for Cancer/Cancer Hospital, Chinese Academy of Medical Sciences and Peking Union Medical College, 17 Pan-jia-yuan South Lane, Chaoyang District, Beijing, 100021 China; 2 Department of Thoracic/Head & Neck Medical Oncology, UT MD Anderson Cancer Center, Houston, TX 77030 USA; 3 Department of Oncology, Tongji Hospital, Tongji Medical College, Huazhong University of Science and Technology, 1095 Jiefang Ave. Qiaokou District, Wuhan, 430030 Hubei China; 4 Department of Thoracic Surgery, National Cancer Center/Cancer Hospital, Chinese Academy of Medical Sciences and Peking Union Medical College, Beijing, China; 5 State Key Laboratory of Oncology in South China, Collaborative Innovation Center for Cancer Medicine, Sun Yat-sen University Cancer Center, 651# East Dong Feng Road, Guangzhou, 510060 Guangdong China; 6 School of Pharmaceutical Sciences, Xiamen University, Xiamen, 361102 Fujian China

**Keywords:** TMB estimation, 23-gene panel, Prognostic and predictive value, Non-small cell lung cancer

## Abstract

**Background:**

Tumor mutational burden (TMB) has both prognostic value in resected non-small cell lung cancer (NSCLC) patients and predictive value for immunotherapy response. However, TMB evaluation by whole-exome sequencing (WES) is expensive and time-consuming, hampering its application in clinical practice. In our study, we aimed to construct a mutational burden estimation model, with a small set of genes, that could precisely estimate WES-TMB and, at the same time, has prognostic and predictive value for NSCLC patients.

**Methods:**

TMB estimation model was trained based on genomic data from 1056 NSCLC samples from The Cancer Genome Atlas (TCGA). Validation was performed using three independent cohorts, including Rizvi cohort and our own Asian cohorts, including 89 early-stage and *n* late-stage Asian NSCLC patients, respectively. TCGA data were obtained on September 3, 2018. The two Asian cohort studies were performed from September 1, 2018, to March 5, 2019. Pearson’s correlation coefficient was used to assess the performance of estimated TMB with WES-TMB. The Kaplan-Meier survival analysis was applied to evaluate the association of estimated TMB with disease-free survival (DFS), overall survival (OS), and response to anti-programmed death-1 (PD-1) and anti-programmed death-ligand 1 (PD-L1) therapy.

**Results:**

The estimation model, consisted of only 23 genes, correlated well with WES-TMB both in the training set of TCGA cohort and validation set of Rizvi cohort and our own Asian cohort. Estimated TMB by the 23-gene panel was significantly associated with DFS and OS in patients with early-stage NSCLC and could serve as a predictive biomarker for anti-PD-1 and anti-PD-L1 treatment response.

**Conclusions:**

The 23-gene panel, instead of WES or the currently used panel-based methods, could be used to assess the WES-TMB with a high relevance. This customized targeted sequencing panel could be easily applied into clinical practice to predict the immunotherapy response and prognosis of NSCLC.

## Background

Tumor mutational burden (TMB), commonly defined as the number of nonsynonymous mutations, has been proposed as a promising predictive biomarker for the response to immune checkpoint inhibitors (ICIs). Importantly, this metric tightly correlates with overall survival (OS) in resected non-small cell lung cancer (NSCLC) patients [1]. In 2015, Rizvi et al. demonstrated that an increased number of nonsynonymous mutations were associated with improved objective response, durable clinical benefit (DCB), and progression-free survival (PFS) in NSCLC patients who received anti-programed death (PD)-1 therapy [2]. Clinical studies have also revealed a significant correlation between TMB and objective response rate (ORR) to ICIs in multiple tumor types [3–5]. In addition, Devarakonda et al. recently reported that high TMB was associated with a better survival prognosis in patients with resected NSCLC, and the benefit of adjuvant chemotherapy was more pronounced in patients with low TMB [6].

The gold standards for TMB calculation are through whole-genome sequencing (WGS) or whole-exome sequencing (WES). However, several obstacles, such as the high demand for quality and quantity of tissue samples, the cost and time consumption, and the unavailability for translation to TMB evaluation by circulating tumor DNA (ctDNA) in blood (bTMB) [7], hinder the clinical application of these techniques. As a result, targeted next generation sequencing (NGS) of cancer-related gene panels (CGP) has been developed, serving as surrogates for WES for TMB estimation. To date, the Food and Drug Administration (FDA) has approved several NGS panels for TMB estimation (e.g., FoundationOne CDx (F1CDx) and Memorial Sloan Kettering Cancer Center’s Integrated Mutation Profiling of Actionable Cancer Targets (MSK-IMPACT)), which include about 300–500 genes and cover over one megabase of coding DNA [7, 8]. Recently, many new NGS panels consisting of different numbers of genes have been developed and validated, most of which were designed initially for guiding the use of target therapies. These panels mainly include cancer-related oncogenes and tumor suppressor genes, many of which do not contribute to or even negatively correlate with TMB, thus are not accurate for TMB evaluation. Besides, inclusion of these genes in an NGS panel enlarges the panel size used for TMB estimation and can lead to an inferior cost-effective consequence. It is important to note that cancer type-specific mutation load estimation models have proven to be necessary because of the different mutation landscapes among varying tumor types [9]. Although DNA damage repair (DDR) genes, negatively predictive genes (*STK11* and *KEAP1*), and TMB-associated genes such as *MUC16*, *POLE*, *POLD1*, and *TTN* have been included in the NGS panels for TMB evaluation [10–14], with the burgeoning developments in immunotherapy, there is a need for more specific panels that focus on TMB estimation for NSCLC.

Herein, by using The Cancer Genome Atlas (TCGA) database as a training set and multiple real-world cohorts as a validation set, we constructed an optimized TMB estimation model with the smallest number of carefully selected TMB-associated genes that could be used as both predictive markers for immunotherapy and prognosis biomarkers for resected NSCLC patients.

## Methods

### Patient cohorts

Genomic and clinical data for 1026 NSCLC samples, including 522 lung adenocarcinoma (LUAD) and 504 lung squamous cell carcinoma (LUSC) samples, were downloaded from TCGA database for the model construction. For the validation of the model, three independent cohorts were used, including a previously published study (the Rizvi cohort [2]), a surgery cohort composing of 89 early-stage NSCLC patients who underwent surgical treatment, and a ZS immunotherapy cohort composing of 73 advanced NSCLC patients who received ICI treatment. All the 73 patients in the ZS immunotherapy cohort received either anti-PD-1 (nivolumab, *n* = 14; pembrolizumab, *n* = 35; SHR-1210, *n* = 19) or anti-PD-L1 (atezolizumab, *n* = 5) monotherapy agents. There are 34 patients who received durable clinical benefit (DCB, anti-PD-1 (*n* = 31), anti-PD-L1 (*n* = 3)) and 39 patients with no durable benefit (NDB, anti-PD-1 (*n* = 37), anti-PD-L1 (*n* = 2)). All three validation cohorts were used to evaluate the performance of the TMB estimation model. Additionally, the surgery cohort was also used for survival validation in resected NSCLC patients. Both the Rizvi and immunotherapy cohorts were also used for validation of ICI outcome predictability in advanced NSCLC patients. The clinical details for all enrolled patients were collected. The treatment efficacy for those treated with immunotherapy was assessed using Response Evaluation Criteria in Solid Tumors (RECIST) version 1.1, with durable clinical benefit (DCB) defined as partial or stable disease lasting over 6 months [15]. All procedures were approved by the ethics committees of the National Cancer Center. All patients provided written informed consent.

### Whole-exome sequencing and data processing

We performed whole-exome sequencing of samples from two cohorts in the validation set, including 89 early-stage NSCLC patients who underwent surgical treatment and 73 advanced NSCLC patients who received ICI treatment. For those 89 early-stage NSCLC patients, both tumor and matched normal samples were obtained and subjected to WES. Briefly, DNA libraries were prepared using the MGIEasy Exome Capture V4 Probe Set capture kit (cat. no: 1000007745) with a capture region size of 36 Mb. BGI-Seq 500 instruments were used for pair-end sequencing (2 × 100 bp). The data were processed according to the manufacturer’s protocol [16]. The mean coverage was 167× and 161× in tumor and normal samples, respectively.

For those 73 advanced NSCLC patients, biopsy specimens were available for WES. The genomic DNA was extracted using the QIAamp DNA FFPE Tissue Kit and quantified using the dsDNA HS Assay Kit (ThermoFisher Scientific, USA). Libraries were constructed with the KAPA Hyper Prep Kit (KAPA Biosystems, USA). An Illumina HiSeq4000 platform was used for sequencing with PE150 sequencing 161 chemistry (Illumina, USA) [17]. The average coverage depth was 140×.

### Candidate gene selection

Genomic data for 1026 NSCLC samples from TCGA were used for candidate gene selection, which were used to construct the mutation load estimation model. The candidate genes were selected based on two criteria: mutation frequency higher than or equal to 10% and significant association with mutation load [9]. The mutation frequency of a gene was calculated as the percentage of patients with mutation in the gene. Mutation load-associated genes were defined as where the WES-TMB was significantly different between the patients with the mutated gene and those with wild-type counterparts (Additional file 1: Table S1).

### Mutation estimation model construction

The mutation estimation model construction was based on TCGA data in the training set. In detail, the first step was to build a mutation estimation model, using the fewest genes, which tightly associated with WES-TMB. In our study, we constructed the estimation model by simply randomly selecting a specified number of genes from all the genes or TMB-associated genes and summed the mutational number as the estimated TMB. Under every given number of genes, the procedure was repeated 1000 times, resulting in 1000 random models. We then calculated the Pearson correlation coefficient (*r*) between the estimated and actual mutation load of WES-TMB. The results allowed us to select the model with highest *r* under the specified number of genes. The next step was to identify which of those best models under the specified number of genes correlated with the clinical outcomes of overall survival (OS) and disease-free survival (DFS). The final step was to select a model using the fewest genes that tightly associate with the WES-TMB and have both prognostic value for those early-stage NSCLC patients and predictive value for those late-stage NSCLC patients who received ICI treatment.

### RNA expression difference between TMB high and low groups

To compare gene expression patterns, we downloaded an mRNA data set of 1026 NSCLC patients from TCGA database. mRNA expression was analyzed using gene set enrichment analysis (GSEA) (http://software.broadinstitute.org/gsea/index.jsp) [18]. We divided these patients into estimated high (≥ 4 mutational counts) and low TMB groups (< 4 mutational counts), and identified whether immune-related gene signatures associated with tumor mutation status. The genes found to be on the leading edge of the enrichment profile were subjected to pathway analysis. Genes with expression over 0 in more than 80% of the samples were included in the GSEA. The normalized enrichment score (NES) is generally the primary statistic for examining gene set enrichment results.

### Statistical analysis

The Mann-Whitney *U* test was used to assess the differences in the mutation load between the two groups. The genes with Kruskal-Wallis-corrected *p* values lower than 0.05 were identified as the mutation load-associated genes and selected as potential candidate genes. Survival analysis was performed using the Kaplan-Meier curves, with a *p* value determined by a log-rank test, and the statistical tests were two-sided and considered statistically significant at *p* < 0.05, unless otherwise stated. The analyses were performed using GraphPad Prism version 5.0 (GraphPad Prism). Correlations between estimated mutation burden and whole-exome sequencing-calculated TMB were determined by Pearson’s correlation coefficient. The analyses were performed using R-3.5.3.

## Results

### Candidate gene selection for model construction

The flowchart of the construction of estimation model is shown in Fig. S1 in Additional file 1. The somatic mutation data of 1026 cases of NSCLC were downloaded from TCGA database as the training set (TCGA cohort), including 522 adenocarcinoma and 504 squamous cell carcinoma subtypes of NSCLC (Additional file 1: Table S2). Subsequently, a mutation matrix including screened nonsynonymous mutations in 181,115 genes was generated. Furthermore, we identified genetic alterations in 116 genes with mutation frequency ≥ 10% in general NSCLC patients and significantly correlating with WES-TMB (*p* value range 6.95E−54 to 4.52E−3). These 116 genes were then used as candidate genes for the construction of the TMB estimation model (Additional file 1: Table S3).

### Construction of the TMB estimation model

**Fig. 1:**
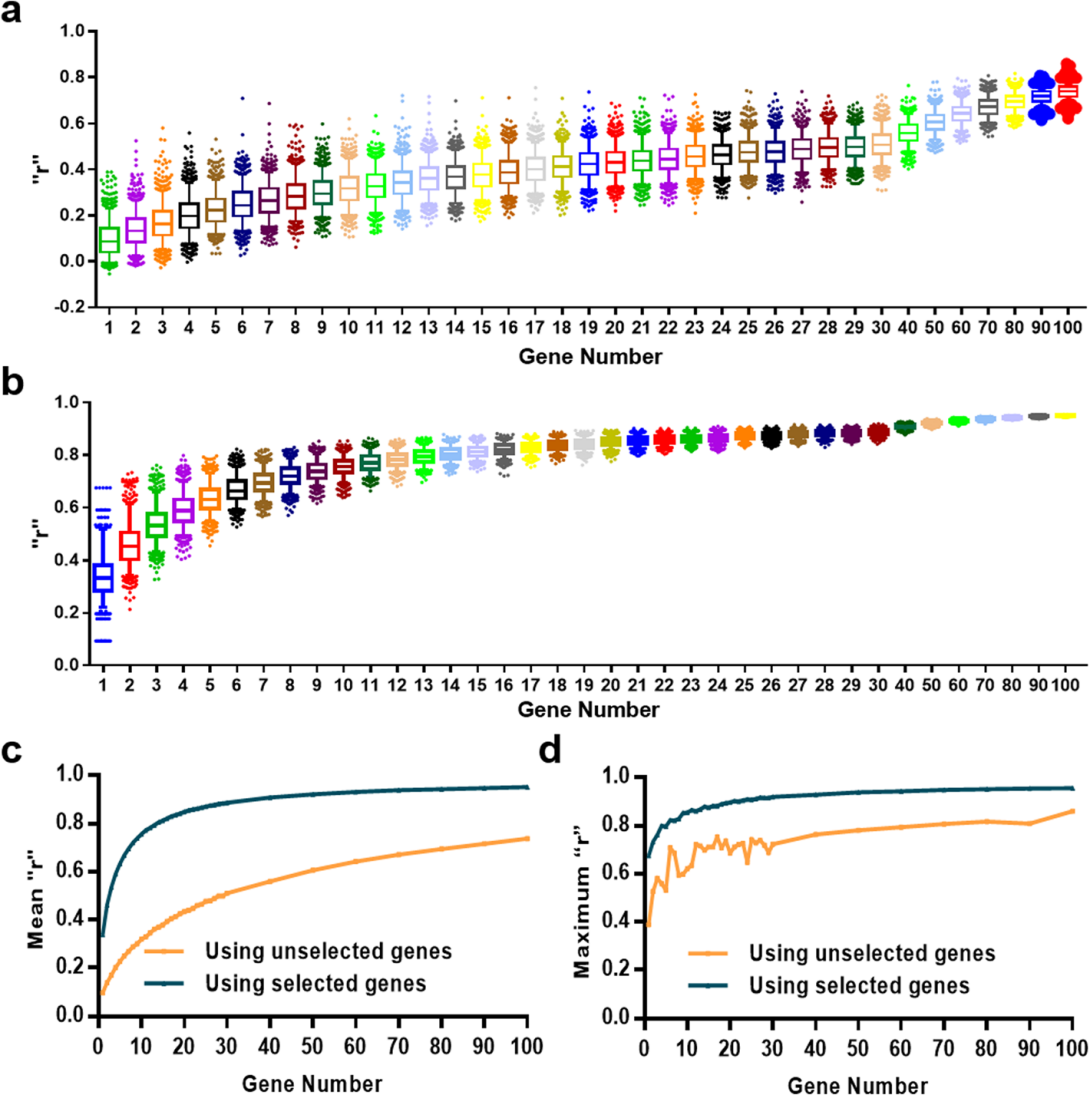
The correlation of WES-TMB and TMB as estimated by different gene panels. **a**, **b** Correlation is represented by the Pearson correlation coefficient (*r*). Genes used for the mutation model construction were either from unselected genes (**a**) or from 116 selected genes (**b**) that correlate with WES-TMB. **c**, **d** Comparisons of mean (**c**) and maximum (**d**) *r* of estimated TMB and WES-TMB, using unselected genes or selected 116 genes

Genes used for the TMB estimation model were randomly selected from the 116 candidate genes, and the estimated TMB was defined as the sum of all nonsynonymous mutation counts of the selected genes. Under each specified number of abstracted genes, the procedure was repeated 1000 times, thus resulting in 1000 separate random models. The serial correlations of estimated TMB by these random models and WES-TMB were evaluated using the Pearson correlation coefficient (*r*). As expected, the correlations between the estimation models and WES-TMB increased with the number of genes (Fig. 1a, b, Additional file 1: Fig. S2a, b). Compared with unselected genes in the range of genomic genes, the estimated TMB based on 116 selected genes was significantly more closely associated with WES-TMB in terms of either the mean or the maximum *r* (Fig. 1c, d, Additional file 1: Fig. S2c, d). The maximum *r* increased from 0.675 with one gene included to greater than 0.900 with 21 genes included and then reached a plateau. When the included gene number exceeded 21, the *r* values were comparable, though increased slowly as the number increased (Fig. 1b). We asserted that *r* greater than 0.9 in the estimation models was acceptable. As such, we considered a model with this effect, but including the least number of genes, an ideal model for clinical application.

**Fig. 2:**
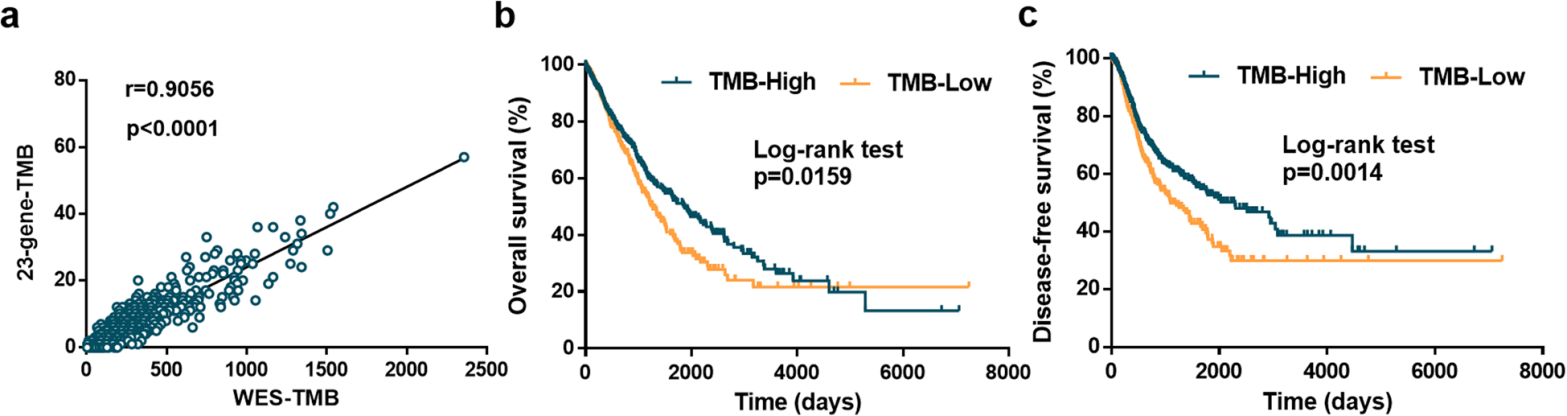
TMB estimation model construction based on TCGA data in the training set. **a** The correlation of 23-gene TMB with WES-TMB is 0.9056, with an empirical *p* value of *r* of *p* < 0.0001. **b** The overall survival is significantly higher in the TMB-high group (≥ 4 mutational counts, *n* = 576) than in the TMB-low group (< 4 mutational counts, *n* = 414), with log-rank test *p* < 0.05. **c** The disease-free survival is significantly higher in the TMB-high group than in the TMB-low group, with log-rank test *p* < 0.01

In reference to previous reports that TMB is associated with prognosis in patients with resected NSCLCs, the optimal TMB estimation model was further evaluated based on the correlation of estimated TMB with OS and DFS in models with *r* over 0.9. Ultimately, we constructed an estimated TMB model with only 23 genes and *r* of 0.9056 (*p* < 0.0001; Fig. 2a, Additional file 2), which was significantly associated with both OS and DFS (Fig. 2b, c). The cutoff value of the estimated TMB by the 23-gene panel was defined as 4 mutational counts (the median value of estimated TMB based on TCGA database) (Additional file 1: Fig. S3a, b) that were equal or over 4 mutational counts as TMB-high cases and less than 4 mutational counts as TMB-low ones. These genes included *UNC13C*, *HMCN1*, *ZNF536*, *KMT2D*, *USH2A*, *XIRP2*, *PCDH15*, *AHNAK2*, *ADGRL3*, *RELN*, *NF1*, *TTN*, *ADGRG4*, *CUBN*, *CACNA1E*, *MRC1*, *COL11A1*, *NAV3*, *CSMD1*, *APOB*, *CSMD3*, *COL22A1*, and *EPHA5* (Additional file 1: Table S4). The model yielded good performances in both subtypes of NSCLC, with correlations of 0.9244 for LUAD (Additional file 1: Fig. S4a) and 0.8781 for LUSC (Additional file 1: Fig. S4b). The average CDS length of these 23 genes was 12k nucleotides (3k–80k, Additional file 1: Table S4), and the total length was 0.28M nucleotides, which was considered to be a great reduction of sequencing cost for mutation load estimation. We concluded that the 23-gene panel is the ideal model based on TCGA training set.

### Analytic validation of the 23-gene panel in Asian resected NSCLC patients

**Fig. 3:**
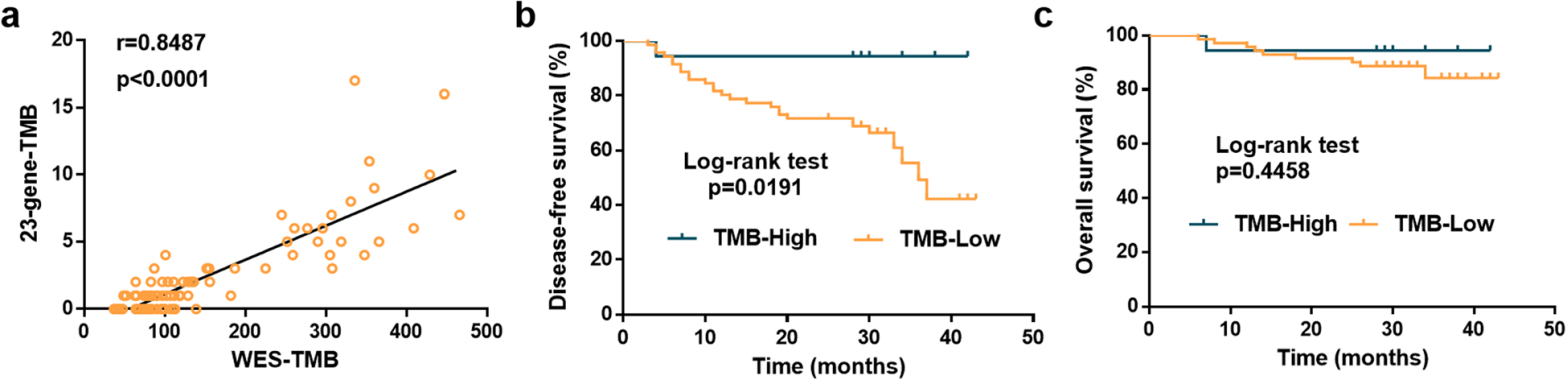
Validation of the TMB estimation model based on the 89 early-stage NSCLC patients in the validation set. **a** The Pearson correlation coefficient of estimated TMB by the 23-gene panel and WES-TMB is 0.8487, with an empirical *p* value of *r* of *p* < 0.0001. **b** The disease-free survival is higher in the estimated TMB-high group (≥ 4 mutational counts, *n* = 18) than in the TMB-low group (< 4 mutational counts, *n* = 71), with log-rank test *p* < 0.05. **c** The overall survival is comparable in the two groups, with log-rank test *p* = 0.4458

To validate the performance of the estimation model, we conducted WES on 89 Chinese, stage IA–IIIA NSCLC patients after radical pneumonectomy (surgery cohort, Additional file 1: Table S1). The correlation of 23-gene TMB with WES was 0.8487 (*r*, *p* < 0.0001, Fig. 3a). As shown in Fig. 3b, TMB-high (≥ 4 mutational counts) according to the 23-gene panel associated with a better DFS compared with those with TMB-low (log-rank, *p* = 0.0191). Besides, a tendency towards improved OS was observed in the patients with higher estimated TMB, though a statistical difference was not reached due to the fact that most patients were still alive (Fig. 3c).

### Performance verification by comparing the 23-gene panel with other commercial panels

**Fig. 4:**
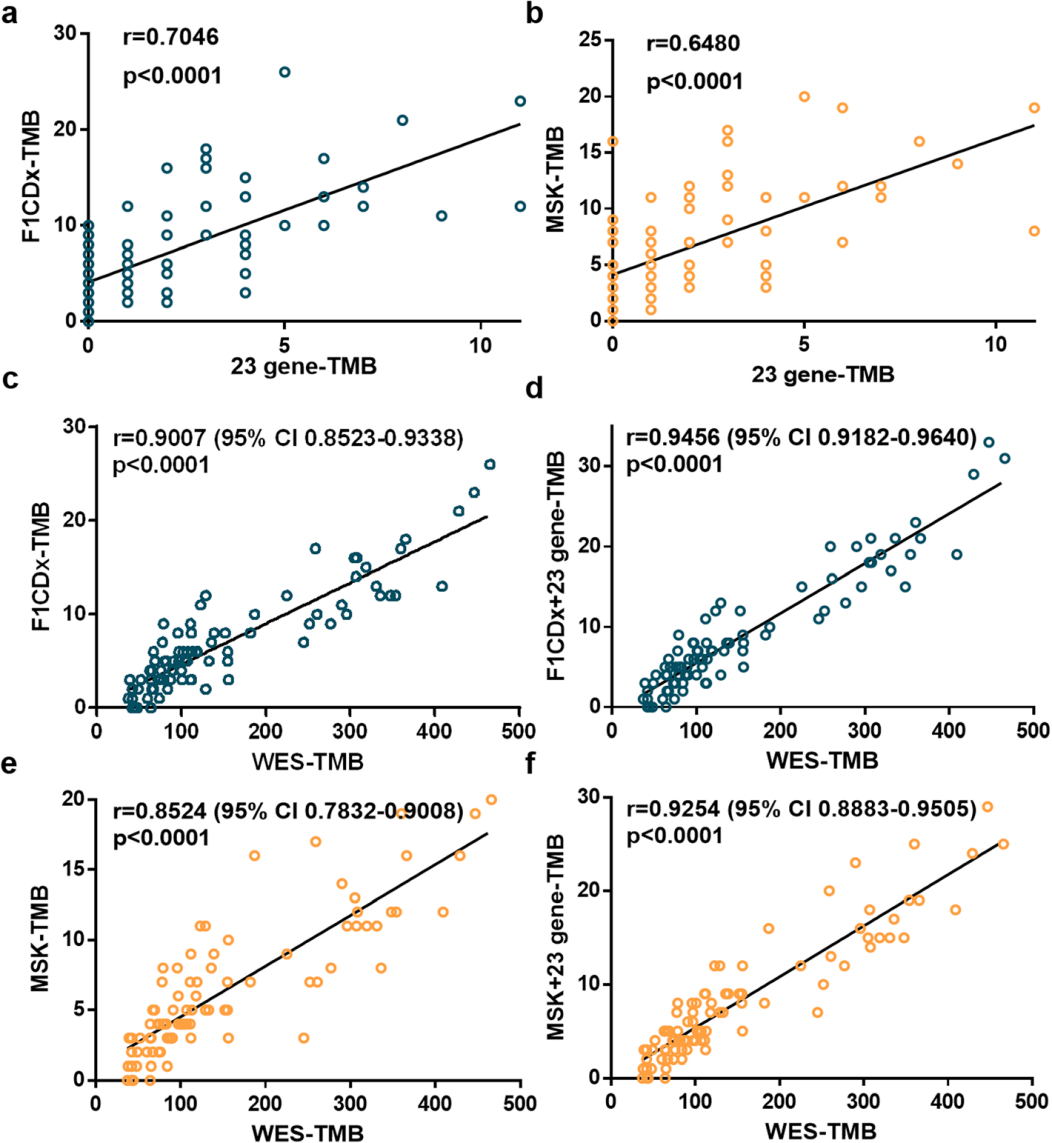
Performance evaluation of the 23-gene panel against commercially used gene panels. **a**, **b** The Pearson correlation coefficient of 23-gene TMB with F1CDx-TMB (**a**) and MSK-TMB (**b**). **c**, **d** The Pearson correlation coefficient of WES-TMB with F1CDx-TMB (**c**) and incorporated panel of 405 cancer-associated genes in F1CDx with 23-gene panel (F1CDx + 23 gene-TMB) (**d**). **e**, **f** The Pearson correlation coefficient of WES-TMB with MSK-TMB (**e**) and incorporated panel of 414 cancer-associated genes in MSK-IMPACT with 23 gene-panel (MSK + 23 gene-TMB) (**f**)

Next, we compared the 23-gene panel with two commercial panels based the 73 early-stage NSCLC data, including F1CDx (405 genes) and MSK-IMPACT (414 genes). There are two overlap genes between the 23-gene panel with F1CDx and MSK-IMPACT, namely *NF1* and *EPHA5*. The 23-gene TMB has a tight correlation with the TMB estimated by F1CDx (F1CDx-TMB) or MSK-IMPACT (MSK-TMB) (*r* = 0.7046 and 0.6480, respectively, both *p* < 0.0001, Fig. 4a, b). In addition, when the 23 genes were added to the two commercial panels, the correlation of the incorporated panels with WES-TMB significantly increased from 0.9437 (95% CI 0.8523–0.9338) to 0.9579 (95% CI 0.9182–0.9640) (*p* < 0.05) for F1CDx (Fig. 4c, d) and from 0.9270 (95% CI 0.7832–0.9008) to 0.9579 (95% CI 0.8883–0.9505) (*p* < 0.05) for MSK-IMPACT (Fig. 4e, f). To further verify the specificity of these 23-gene panels, we compared them with other 23 randomly selected gene panels from the 116 genes. The procedure was repeated 1000 times, resulting in the random Pearson correlation coefficients from 0.8958 to 0.9455 of F1CDx plus random 23 genes and from 0.8642 to 0.9291 of MSK plus random 23 genes. The performance of our 23-gene model was better than 99% of random models, which indicated the irreplaceability of these genes.

Based on the survival data from our 89 early-stage NSCLC patients, significant correlations were observed between survival outcomes (DFS) and the TMB level stratified with F1CDx or MSK-IMPACT panel (Additional file 1: Fig. S5a, c). Interestingly, the 23 genes could improve the association of these two commercial panels with DFS (Additional file 1: Fig. S5b, d). If the incorporated panels were used for analysis, TMB-high estimated by both of the two new panels (F1CDx + 23-gene panel or MSK-IMPACT + 23-gene panel) demonstrated improved DFS compared with those of estimated TMB-low under the cutoff values indicated in Fig. S3c and S5 of Additional file 1.

### Immune-regulatory gene expression signatures stratified by TMB level based on the 23-gene panel

**Fig. 5:**
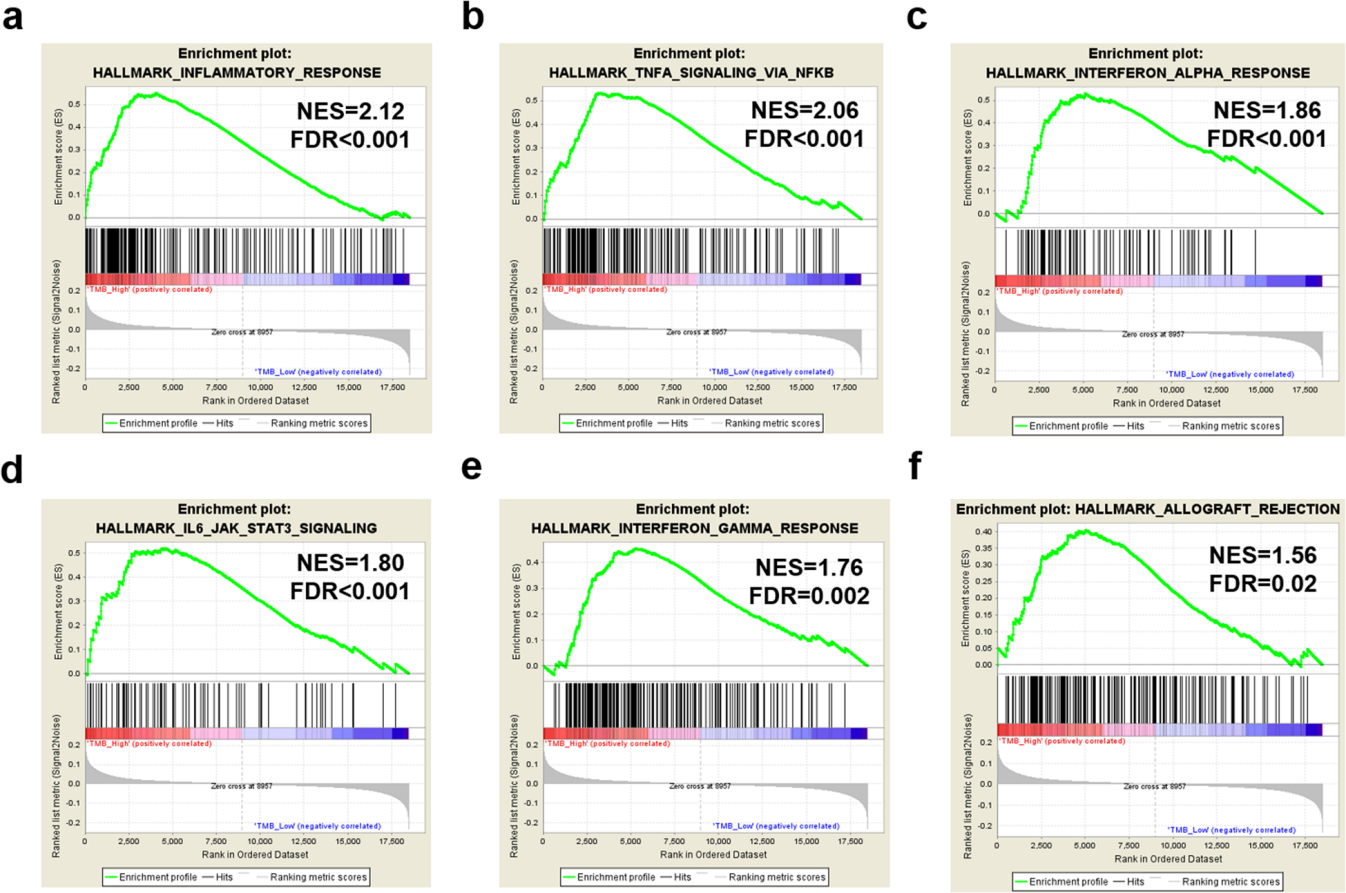
Gene expression differences between the estimated high TMB and low TMB groups. **a**–**f** TMB-associated pathways, such as inflammatory response, TNF-α signaling via NFκB, interferon α response, IL6-JAK-STAT3 signaling, interferon γ response, and allograft rejection. NES, normalized enrichment score; FDR, false discovery rate

To investigate the difference in immune status between TMB-high and TMB-low estimated by the 23-gene panel, we analyzed immune-regulatory gene expression signatures based on the RNAseq data of 1026 NSCLC cases from TCGA. The GSEA revealed a prominent enrichment of mRNA signatures involved in the inflammatory response; TNF-α; interferon-α, γ (IFN-α, γ) response; IL6-JAK-STAT3 signaling; and allograft rejection (Fig. 5).

### Immunotherapy response prediction by the established 23-gene panel

**Fig. 6:**
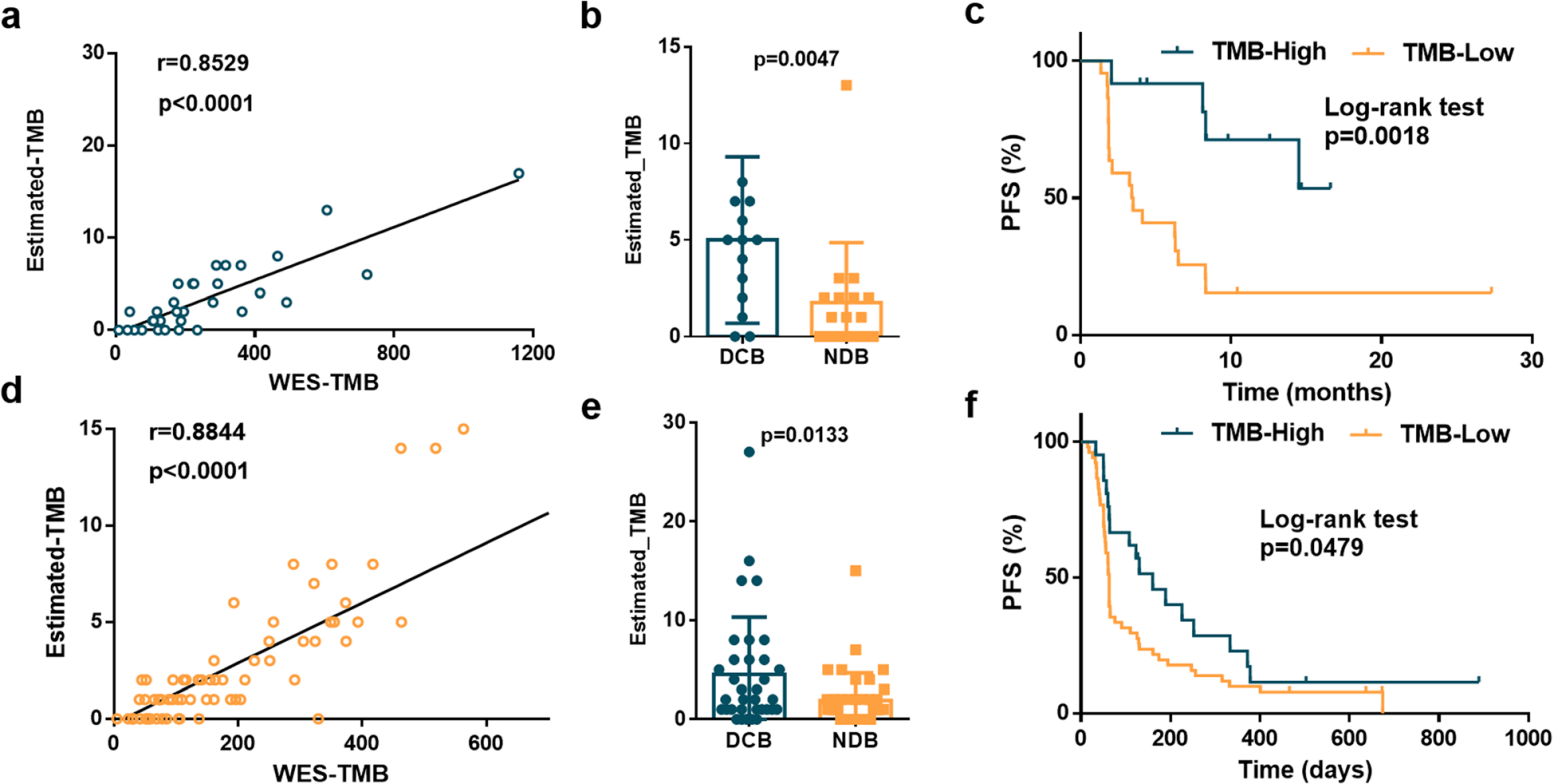
Immunotherapy response estimation by the 23-gene panel. **a** The correlation of the estimated TMB with WES-TMB using the Rizvi data (*n* = 34). **b** Estimated TMB in tumors from patients with DCB (*n* = 14) or with NDB (*n* = 17; Mann-Whitney, *p* = 0.0047). **c** PFS in tumors with estimated TMB-high (*n* = 12) compared to tumors with TMB-low (*n* = 22) in patients in the Rizvi cohort (HR 0.39, 95% CI 0.16 to 0.86, log-rank *p* = 0.0018). **d** The correlation of estimated TMB with WES-TMB using the late-stage NSCLC patient cohort (*n* = 73). **e** Estimated TMB in tumors from patients with DCB (*n* = 34) or with NDB (*n* = 39; Mann-Whitney, *p* = 0.0133). **f** PFS in tumors with estimated TMB-high (*n* = 21) compared to tumors with TMB-low (*n* = 52) in patients in the late-stage NSCLC patient cohort (HR 0.40, 95% CI 0.26 to 0.94, log-rank *p* = 0.0479)

Finally, we analyzed the performance of TMB estimated by the 23-gene panel in the prediction of response to ICIs, using two independent NSCLC cohorts. In the Rizvi cohort, the correlation between the TMB estimated by the 23-gene panel and WES was 0.8529 (empirical *p* value of *r* < 0.0001, Fig. 6a). The estimated TMB was significantly different between the patients with durable clinical benefit (DCB; a partial or stable response lasting over 6 months) and no durable benefit (NDB; Mann-Whitney *p* = 0.0047; Fig. 6b). Survival analysis was then applied for the comparison of the PFS between the patients (*n* = 34) with TMB-high (≥ 4 counts) and TMB-low (< 4 counts) by the 23-gene panel. Patients with TMB-high demonstrated significantly improved PFS compared with those with TMB-low (14.5 vs. 3.5 months, log-rank *p* = 0.0238) (Fig. 6c).

To further validate the performance of the estimation model for response to ICIs, we performed WES of 73 advanced (stage IIIB–IV) NSCLCs in another Asian cohort (ZS immunotherapy cohort). All of these 73 patients received with anti-PD-1 or anti-PD-L1 treatment. The *r* between the estimated and actual mutation burden was calculated to be 0.8844 (empirical *p* value of *r* < 0.0001, Fig. 6d). The estimated TMB was significantly different between the patients with DCB and NDB (Mann-Whitney *p* = 0.0133, Fig. 6e). The PFS was associated with estimated TMB (log-rank *p* = 0.0479, Fig. 6f), demonstrating that the estimated mutation burden derived from Caucasian NSCLCs from TCGA could predict the immunotherapy treatment response quite well in Asian patients. We further calculated the HR at different cutoff values in the ZS immunotherapy cohort and found the 3 mutational counts in this cohort resulted the best HR value (Additional file 1: Fig. S6). As a result, when applied in clinical practice, the cutoff value still needs to be further evaluated accordingly.

### Comparison of the 23-gene panel with previously reported TMB-related genes

Mutations in *TTN*, *MUC16*, *POLE*, and *POLD1* have been previously reported to correlate with elevated TMB levels [12–14]. The frequencies of these 4 genes in NSCLC, based on 1026 cases from TCGA, were 46%, 40%, 4.8%, and 0%, respectively. WES-TMB was significantly different between the patients with these mutated genes and those with wild-type counterparts (Additional file 1: Fig. S7). However, only *MUC16* mutations exhibit significant correlation with OS and DFS in TCGA cohort (Additional file 1: Fig. S7a-c), while they failed to confirm the results in our surgery cohort (Additional file 1: Fig. S8). Notably, none of these 3 gene mutations could predict the response or PFS in either the Rizvi cohort or our immunotherapy cohort (Additional file 1: Fig. S9).

## Discussion

In the present study, we developed a novel and optimal TMB estimation model composed of only 23 genes, which allowed precise estimation of the WES-based TMB both in early-stage and late-stage NSCLC patients. Importantly, our established 23-gene panel can successfully predict the survival outcomes in both resected NSCLCs and patients receiving ICIs in multiple validation cohorts. To the best of our knowledge, our TMB estimation model is both the first and the smallest panel described to date, which can be used as a biomarker to stratify patients not only after radical pneumonectomy, but also with advanced NSCLC receiving ICIs.

The total CDS length of the 23-gene panel was 0.28M nucleotides, with an average of 12k (3k–80k). The *TTN* is also included in our panel; although it has the longest CDS length of 81k, the total length was acceptable when *TTN* is included. Besides, in a recent study, *TTN* mutation was reported to be associated with TMB in solid tumors, including NSCLC, and correlated with response to ICIs [14]. As a result, the 23-gene panel was considered to be a great reduction of sequencing cost for mutation load estimation.

Several cancer-related genes have been previously reported to be associated with WES-TMB in some cancer types. For example, melanoma patients with *LRP1B* mutations exhibited a higher mutational load than those with the wild-type gene [19]. Li et al. reported that mutations in *MUC16* are associated with TMB and survival outcomes in patients with gastric cancer [13]. Two DDR-related genes (*POLE* and *POLD1*) were also shown to correlate well with WES-TMB in pan-cancer types [12]. Undoubtedly, it would be ideal to utilize a single gene to estimate TMB and effectively predict response to immunotherapy. However, we found that singly, all these genes failed to correlate well with the efficacy of ICIs or survival outcomes after resection; the correlation of any individual gene with WES-TMB was moderate (mean *r* = 0.34 (0.09–0.68)). These results indicate that using a single gene to estimate TMB is insufficient.

Theoretically, the larger a NGS gene panel, the closer the estimated TMB is to the actual amount. However, the cost-effective balance for clinical usage must be considered. In particular, when TMB is detected using peripheral blood, super sequencing depth (e.g., 10,000–20,000×), due to the low abundance of circulating tumor DNA, will significantly drive up the cost [20]. To date, two commercial gene panels (F1CDx and MSK-IMPACT) have been widely used for TMB estimation. These two panels demonstrate good performance in correlation with WES-TMB [21]. Our established gene panel, which includes a very limited number of genes, demonstrated comparable correlation coefficients with these two large panels, indicating the promising reliability of a small panel as a surrogate for WES-TMB. Notably, the majority of genes used in our model were not included in the currently used commercial gene panels. If the genes in our panel were incorporated into the big commercial gene panels, the correlation coefficients with WES-TMB increased. These results demonstrate that the 23 genes we have selected here may be used independently or as complement to the currently used gene panels specific for NSCLC. Inclusion of the 23 genes should be considered in future NGS gene panels.

Recently, Lyu et al. developed a small gene panel with 24 genes to estimate actual TMB, derived from 230 LUADs in TCGA database. The construction and validation cohorts used for Lyu et al.’s 24-gene panel were mainly from Caucasian patients. However, our 23-gene panel, though also derived from TCGA database, was successfully validated in multiple Asian patient cohorts. These results suggest that our 23-gene panel may be more suitable to NSCLC and applicably potent regardless of race and subtypes (Additional file 1: Fig. S10).

Similar with the findings of Devarakonda et al. [6], we observed that high TMB associated with improved OS in resected NSCLC patients. In colon cancer patients with resected stage II, mismatch repair deficiency, high TMB has been utilized as a good prognostic biomarker [22]. Indeed, these results possess internal rationality. Both high neo-epitope burden [23] and intense TIL infiltration [24] have been associated with favorable survival outcomes in early-stage lung cancer. High TMB may reflect the immunogenicity in some degree, which could mediate the shaping of tumor-host immune interactions. Taken together, these and our findings suggest that quantifying genomic instability through TMB estimation can be used to stratify patients so as to guide adjuvant treatment.

Owing to the lack of information on HLA-I, it is difficult to judge whether the predictive value of our gene panel is due to neo-antigen generation derived from the included gene mutations or if the estimated TMB based on the 23-gene panel is simply a representative reflection of genomic instability as an “accompanying passenger.” The other limitation of our study is the small number of patients who received the immunotherapy treatment. Thus, a larger number of cases from a multicenter study are required for the validation of the performance of the treatment response prediction. In addition, our validation cohorts were retrospective; a prospective study is necessary to translate our estimation model into clinical practice. In addition to TMB, other features, such as PD-L1 expression, microsatellite instability, and neo-antigen burden, have emerged as potential predictive biomarkers for ICIs [25–27]. However, challenges in defining cutoff values, intertumoral and intratumoral heterogeneity, and test platform uniformities have limited their clinical applications [10]. Therefore, future strategies that combine different predictive features may be more effective biomarkers for the accurate prediction of cancer immunotherapy response [28], but need to be carefully integrated.

## Conclusions

In summary, we have successfully constructed a novel TMB estimation model using only 23 genes that can be used to estimate the WES-TMB, and stratify survival prognosis after radical surgery and clinical outcomes of ICI therapy in NSCLC patients. Thus, a customized panel for the targeted sequencing of these selected genes, instead of whole-exome sequencing, can be designed or utilized as complementary genes included in the current NGS panels. Consequently, by using our model, the cost-effectiveness may be considerably improved, making realization of cancer immunotherapy response more accessible in standard clinical settings [29].

## Supplementary information

**Additional file 1: Table S1.** Data sets used to calculate WES-TMB for the 4 study cohorts. **Table S2.** Characteristics of the patients included in this study. **Table S3.** 116 candidate genes and related information. **Table S4**. 23 genes and the corresponding CDS length. **Fig. S1.** Flowchart of the construction of estimation model. **Fig. S2.** The correlation of WES-TMB and TMB, as estimated by different gene panels. **Fig. S3.** Forest plots of HRs for OS and DFS in the TCGA and 89 early-stage NSCLC patients study cohort. **Fig. S4.** The performance of 23-gene based TMB estimation model for the LUAD and LUSC subtypes of NSCLC (TCGA data). **Fig. S5.** Forest plots of HRs for DFS in the 89 early-stage NSCLC patients study cohort. **Fig. S6.** Forest plots of HRs for PFS of the 73 NSCLC patients in ZS immunotherapy cohort. **Fig. S7.**. WES-TMB is shown based on MUC16 (a), TTN (b) and POLE (c) mutation status. **Fig. S8.** The correlation of MUC16 mutation status with overall survival (a) and disease-free survival (b) based on the 89 early-stage NSCLC patients. **Fig. S9.** The correlation of MUC16, TTN and POLD1 mutation status with progression-free survival (PFS) based on the *Rizvi* cohort and our immunotherapy cohort. **Fig. S10.** Comparison of predictive performance of response to ICIs by our 23-gene panel with Lyu’s 24-gene panel.

**Additional file 2.** The correlation of estimation models with gene number (1 to 30) with OS and DFS in the training set.

## Data Availability

Data are available in the publications cited in the manuscript.
