# Supplementary material for: A novel tumor mutational burden estimation model as a predictive and prognostic biomarker in NSCLC patients
Source: BMC Med. 2020 Aug 26;18:232. doi: 10.1186/s12916-020-01694-8 (PMC7448445; doi:10.1186/s12916-020-01694-8)
Supplement: Supplementary file 1 — Additional file 1: Table S1. Data sets used to calculate WES-TMB for the 4 study cohorts. Table S2. Characteristics of the patients included in this study. Table S3. 116 candidate genes and related information. Table S4. 23 genes and the corresponding CDS length. Fig. S1. Flowchart of the construction of estimation model. Fig. S2. The correlation of WES-TMB and TMB, as estimated by different gene panels. Fig. S3. Forest plots of HRs for OS and DFS in the TCGA and 89 early-stage NSCLC patients study cohort. Fig. S4. The performance of 23-gene based TMB estimation model for the LUAD and LUSC subtypes of NSCLC (TCGA data). Fig. S5. Forest plots of HRs for DFS in the 89 early-stage NSCLC patients study cohort. Fig. S6. Forest plots of HRs for PFS of the 73 NSCLC patients in ZS immunotherapy cohort. Fig. S7.. WES-TMB is shown based on MUC16 (a), TTN (b) and POLE (c) mutation status. Fig. S8. The correlation of MUC16 mutation status with overall survival (a) and disease-free survival (b) based on the 89 early-stage NSCLC patients. Fig. S9. The correlation of MUC16, TTN and POLD1 mutation status with progression-free survival (PFS) based on the Rizvi cohort and our immunotherapy cohort. Fig. S10. Comparison of predictive performance of response to ICIs by our 23-gene panel with Lyu’s 24-gene panel. [file 12916_2020_1694_MOESM1_ESM.docx]

**Additional file 1**

**A Novel Tumor Mutational Burden Estimation Model as a Predictive and Prognostic Biomarker in NSCLC Patients**

Yanhua Tian^1,2 †^, Jiachen Xu^1†^, Qian Chu^3†^, Jianchun Duan^1^, Jianjun Zhang^2^, Hua Bai^1^, Zhenlin Yang^4^, Wenfeng Fang^5^, Liangliang Cai^6^, Rui Wan^1^, Kailun Fei^1^, Jie He^4^, Shugeng Gao^4^, Li Zhang^5^*, Zhijie Wang^1^*, Jie Wang^1^*

^1^State Key Laboratory of Molecular Oncology, Department of Medical Oncology, National Cancer Center/National Clinical Research Center for Cancer/Cancer Hospital, Chinese Academy of Medical Sciences and Peking Union Medical College, Beijing, China.

^2^Department of Thoracic/Head & Neck Medical Oncology, UT MD Anderson Cancer Center, Houston, TX 77030, USA.

^3^Department of Oncology, Tongji Hospital, Tongji Medical College, Huazhong University of Science and Technology, 1095 Jiefang Ave. Qiaokou District, Wuhan, HuBei, 430030, China

^4^Department of Thoracic Surgery, National Cancer Center/Cancer Hospital, Chinese Academy of Medical Sciences & Peking Union Medical College, Beijing, China.

^5^State Key Laboratory of Oncology in South China, Collaborative Innovation Center for Cancer Medicine, Sun Yat-sen University Cancer Center, Guangzhou, Guangdong, 510060, China.

^6^School of Pharmaceutical Sciences Xiamen University, Xiamen, Fujian, 361102, China.

^†^These authors contributed equally to this work

*Correspondence to:

Prof. Jie Wang, State Key Laboratory of Molecular Oncology, Department of Medical Oncology, National Cancer Center/National Clinical Research Center for Cancer/Cancer Hospital, Chinese Academy of Medical Sciences and Peking Union Medical College, 17 Pan-jia-yuan South Lane, Chaoyang District, Beijing 100021, China. Tel: +86-139-1070-4669; Email: [zlhuxi@163.com](mailto:zlhuxi@163.com)

Prof. Zhijie Wang, State Key Laboratory of Molecular Oncology, Department of Medical Oncology, National Cancer Center/National Clinical Research Center for Cancer/Cancer Hospital, Chinese Academy of Medical Sciences and Peking Union Medical College, Beijing, China, 17 Pan-jia-yuan South Lane, Chaoyang District, Beijing 100021, China. Tel: 86-10-87788029; Email: [Jie_969@163.com](mailto:Jie_969@163.com)

Prof. Li Zhang, Sun Yat-sen University Cancer Center, 651# East Dong Feng Road, Guangzhou, Guangdong, 510060, China. Email: zhangli6@mail.sysu.edu.cn

**Additional file legends**

1. **Additional file 1: Supplementary Tables S1-S4**

Table S1. Data sets used to calculate WES-TMB for the 4 study cohorts.

Table S2. Characteristics of the patients included in this study.

Table S3. 116 candidate genes and related information.

Table S4. 23 genes and the corresponding CDS length.

1. **Supplementary Figures S1-S8**

Fig. S1. Flowchart of the construction of estimation model.

Fig. S2. The correlation of WES-TMB and TMB, as estimated by different gene panels.

Fig. S3. Forest plots of HRs for OS and DFS in the TCGA and 89 early-stage NSCLC patients study cohort.

Fig. S4. The performance of 23-gene based TMB estimation model for the LUAD and LUSC subtypes of NSCLC (TCGA data).

Fig. S5. Forest plots of HRs for DFS in the 89 early-stage NSCLC patients study cohort.

Fig. S6. Forest plots of HRs for PFS of the 73 NSCLC patients in ZS immunotherapy cohort.

Fig. S7. WES-TMB is shown based on MUC16 (a), TTN (b) and POLE (c) mutation status.

Fig. S8. The correlation of MUC16 mutation status with overall survival (a) and disease-free survival (b) based on the 89 early-stage NSCLC patients.

Fig. S9. The correlation of MUC16, TTN and POLD1 mutation status with progression-free survival (PFS) based on the *Rizvi* cohort and our immunotherapy cohort.

Fig. S10. Comparison of predictive performance of response to ICIs by our 23-gene panel with Lyu’s 24-gene panel.

**Additional file 2:**

The correlation of estimation models with gene number (1 to 30) with OS and DFS in the training set.

**1. Supplementary Tables**

**Table S1. Data sets used to calculate WES-TMB for the 4 study cohorts.**

| **Cohorts** | **Data source** | **Patients (No.)** |
| --- | --- | --- |
| TCGA cohort | TCGA database | 1,026 |
| *Rizvi* cohort | Published data | 34 |
| Early-stage patients cohort | WES | 89 |
| ZS immunotherapy cohort | WES | 73 |

**Table S2. Characteristics of the patients included in this study.**

| **Characteristics of the patients** | | | |
| --- | --- | --- | --- |
|  | **Discovery Set** | **Validation Set** | |
|  | **TCGA Cohort**  **(n=1,026)** | **early-stage**  **patients (n=89)** | **Late-stage**  **patients (n=73)** |
| **Median Age (Range)** | 67 (33-90) | 62 (33-80) | 55 (28-73) |
| **Gender - no. (%)** |  |  |  |
| **Male** | 615 (59.94) | 45 (50.6) | 51 (69.9) |
| **Female** | 411 (40.06) | 44 (49.4) | 22 (30.1) |
| **Disease Stage - no. (%)** |  |  |  |
| **Stage I** | 524 (51.07) | 27 (30.3) | 0 |
| **Stage II** | 287 (29.97) | 17 (19.1) | 0 |
| **Stage III** | 170 (16.57) | 45 (50.6) | 1 |
| **Stage IV** | 33 (3.22) | 0 | 72 |
| **Unavailable** | 12 (1.17) | 0 | 0 |
| **Tumor Pathology no. (%)** |  |  |  |
| **LUAD** | 522 (50.88) | 61 (68.5) | 43 (58.9) |
| **LUSC** | 504 (49.12) | 28 (31.5) | 12 (16.4) |
| **Other NSCLC*** | 0 | 0 | 18 (24.7) |

LUAD, lung adenocarcinoma; LUSC, lung squamous carcinoma.

*Other NSCLC includes the combination of LUAD and LUSC, lymphoepithelioma-like carcinoma, large cell carcinoma and [sarcomatoid](javascript:;) [carcinoma](javascript:;).

**Table S3. 116 candidate genes and related information.**

|  | **n1** | **n2** | **Mean1** | **Mean2** | **sd1** | **sd2** | **p_value** | **p_adjust** | **Mutation Freq** |
| --- | --- | --- | --- | --- | --- | --- | --- | --- | --- |
| **TP53** | 377 | 679 | 180.5 | 293.4 | 218.8 | 239.1 | 2.50E-29 | 3.24E-26 | 64.3% |
| **TTN** | 444 | 612 | 146.2 | 330.7 | 128.3 | 267.7 | 3.83E-58 | 6.95E-54 | 58.0% |
| **MUC16** | 636 | 420 | 173.3 | 374.0 | 147.2 | 292.7 | 1.06E-51 | 9.60E-48 | 39.8% |
| **CSMD3** | 638 | 418 | 179.2 | 366.0 | 163.1 | 285.9 | 5.25E-49 | 2.38E-45 | 39.6% |
| **RYR2** | 664 | 392 | 185.9 | 367.0 | 169.7 | 289.1 | 1.77E-44 | 5.36E-41 | 37.1% |
| **LRP1B** | 708 | 348 | 189.6 | 382.3 | 172.5 | 294.7 | 1.92E-45 | 6.98E-42 | 33.0% |
| **USH2A** | 726 | 330 | 183.1 | 407.2 | 145.0 | 317.7 | 8.95E-50 | 5.42E-46 | 31.3% |
| **ZFHX4** | 744 | 312 | 197.4 | 386.1 | 177.5 | 303.3 | 3.19E-37 | 7.25E-34 | 29.5% |
| **SPTA1** | 818 | 238 | 209.7 | 402.3 | 181.6 | 331.6 | 5.01E-27 | 5.06E-24 | 22.5% |
| **XIRP2** | 819 | 237 | 196.6 | 448.6 | 152.3 | 351.7 | 6.06E-39 | 1.57E-35 | 22.4% |
| **SYNE1** | 829 | 227 | 222.4 | 365.3 | 201.5 | 316.1 | 2.92E-18 | 7.36E-16 | 21.5% |
| **FLG** | 834 | 222 | 202.5 | 443.2 | 157.8 | 361.8 | 1.20E-30 | 1.81E-27 | 21.0% |
| **NAV3** | 838 | 218 | 214.6 | 401.4 | 196.6 | 314.4 | 5.49E-27 | 5.25E-24 | 20.6% |
| **RYR3** | 855 | 201 | 209.9 | 437.0 | 185.4 | 332.5 | 5.99E-36 | 1.21E-32 | 19.0% |
| **PCDH15** | 855 | 201 | 207.9 | 445.5 | 174.3 | 351.5 | 1.09E-33 | 1.98E-30 | 19.0% |
| **FAM135B** | 860 | 196 | 215.5 | 418.1 | 186.8 | 346.0 | 2.14E-26 | 1.85E-23 | 18.6% |
| **COL11A1** | 861 | 195 | 211.7 | 436.0 | 182.3 | 346.2 | 1.62E-29 | 2.27E-26 | 18.5% |
| **CSMD1** | 866 | 190 | 219.5 | 406.4 | 188.8 | 353.1 | 1.52E-19 | 4.68E-17 | 18.0% |
| **FAT3** | 867 | 189 | 215.6 | 425.2 | 187.5 | 346.6 | 4.65E-24 | 3.38E-21 | 17.9% |
| **PCLO** | 867 | 189 | 213.5 | 435.0 | 175.7 | 368.4 | 1.03E-22 | 5.49E-20 | 17.9% |
| **CDH10** | 873 | 183 | 221.4 | 404.4 | 190.8 | 355.7 | 5.48E-17 | 1.12E-14 | 17.3% |
| **ADAMTS12** | 874 | 182 | 220.6 | 409.5 | 196.3 | 339.1 | 1.36E-19 | 4.25E-17 | 17.2% |
| **MUC17** | 875 | 181 | 217.1 | 427.4 | 182.1 | 366.9 | 5.33E-21 | 2.11E-18 | 17.1% |
| **PAPPA2** | 875 | 181 | 220.6 | 410.3 | 193.2 | 348.2 | 1.73E-19 | 5.15E-17 | 17.1% |
| **ZNF536** | 877 | 179 | 212.3 | 453.2 | 183.0 | 350.7 | 1.73E-31 | 2.86E-28 | 17.0% |
| **APOB** | 880 | 176 | 210.9 | 464.3 | 175.1 | 366.2 | 3.73E-29 | 4.51E-26 | 16.7% |
| **ANK2** | 881 | 175 | 220.7 | 416.3 | 190.6 | 358.0 | 4.53E-22 | 2.17E-19 | 16.6% |
| **SI** | 886 | 170 | 214.6 | 454.2 | 186.6 | 351.2 | 8.92E-29 | 1.01E-25 | 16.1% |
| **DNAH9** | 886 | 170 | 217.9 | 436.5 | 195.6 | 336.8 | 6.64E-28 | 7.09E-25 | 16.1% |
| **TNR** | 889 | 167 | 221.5 | 421.3 | 205.9 | 316.0 | 2.57E-23 | 1.56E-20 | 15.8% |
| **RELN** | 894 | 162 | 217.5 | 449.5 | 186.2 | 365.3 | 1.48E-25 | 1.22E-22 | 15.3% |
| **PKHD1L1** | 895 | 161 | 214.6 | 467.4 | 174.7 | 386.1 | 1.71E-23 | 1.07E-20 | 15.2% |
| **ERICH3** | 895 | 161 | 227.1 | 397.6 | 219.5 | 283.1 | 1.04E-20 | 3.94E-18 | 15.2% |
| **RYR1** | 895 | 161 | 220.1 | 436.5 | 183.9 | 381.0 | 1.08E-16 | 2.10E-14 | 15.2% |
| **PKHD1** | 897 | 159 | 226.8 | 401.8 | 209.4 | 322.4 | 2.13E-19 | 6.24E-17 | 15.1% |
| **DNAH5** | 899 | 157 | 228.6 | 393.8 | 209.9 | 326.4 | 1.60E-16 | 2.96E-14 | 14.9% |
| **PCDH11X** | 901 | 155 | 225.8 | 412.1 | 212.9 | 306.4 | 9.16E-23 | 5.04E-20 | 14.7% |
| **ZNF804A** | 903 | 153 | 224.1 | 424.5 | 208.3 | 318.7 | 1.96E-22 | 9.91E-20 | 14.5% |
| **FAT4** | 904 | 152 | 221.5 | 441.0 | 189.8 | 373.4 | 1.16E-19 | 3.70E-17 | 14.4% |
| **LRP2** | 906 | 150 | 220.3 | 451.3 | 189.4 | 371.1 | 1.02E-20 | 3.94E-18 | 14.2% |
| **KMT2D** | 906 | 150 | 236.4 | 354.2 | 222.5 | 298.2 | 2.25E-10 | 9.61E-09 | 14.2% |
| **ABCA13** | 907 | 149 | 224.1 | 430.0 | 201.9 | 343.8 | 1.49E-21 | 6.60E-19 | 14.1% |
| **CNTNAP5** | 907 | 149 | 224.7 | 426.0 | 204.3 | 337.4 | 2.26E-16 | 4.01E-14 | 14.1% |
| **KEAP1** | 908 | 148 | 244.0 | 309.3 | 226.7 | 293.8 | 0.001174 | 0.004524 | 14.0% |
| **DNAH8** | 909 | 147 | 228.7 | 404.4 | 209.5 | 332.0 | 1.32E-17 | 2.96E-15 | 13.9% |
| **CSMD2** | 910 | 146 | 220.5 | 456.3 | 199.3 | 339.9 | 6.81E-27 | 6.19E-24 | 13.8% |
| **LRRC7** | 910 | 146 | 220.4 | 456.9 | 189.6 | 372.5 | 4.11E-21 | 1.66E-18 | 13.8% |
| **HMCN1** | 910 | 146 | 220.2 | 458.2 | 192.6 | 362.1 | 2.23E-20 | 8.26E-18 | 13.8% |
| **DMD** | 912 | 144 | 226.6 | 421.3 | 201.6 | 355.7 | 9.64E-18 | 2.27E-15 | 13.6% |
| **COL22A1** | 917 | 139 | 221.6 | 460.8 | 189.5 | 381.3 | 6.06E-24 | 4.08E-21 | 13.2% |
| **ADGRB3** | 918 | 138 | 227.3 | 424.8 | 209.7 | 329.1 | 7.61E-19 | 2.10E-16 | 13.1% |
| **ADGRG4** | 920 | 136 | 229.1 | 415.4 | 213.8 | 318.7 | 1.14E-17 | 2.60E-15 | 12.9% |
| **BRINP3** | 921 | 135 | 235.5 | 373.1 | 228.8 | 265.9 | 8.95E-16 | 1.34E-13 | 12.8% |
| **AHNAK2** | 922 | 134 | 223.6 | 456.2 | 191.9 | 383.7 | 5.30E-20 | 1.72E-17 | 12.7% |
| **CUBN** | 922 | 134 | 227.4 | 430.3 | 204.1 | 353.7 | 5.77E-17 | 1.17E-14 | 12.7% |
| **CACNA1E** | 923 | 133 | 221.0 | 476.0 | 190.7 | 376.9 | 5.01E-23 | 2.85E-20 | 12.6% |
| **PXDNL** | 923 | 133 | 225.7 | 443.5 | 203.9 | 348.0 | 2.21E-22 | 1.09E-19 | 12.6% |
| **FAT1** | 923 | 133 | 231.9 | 400.4 | 208.8 | 352.0 | 2.65E-13 | 2.47E-11 | 12.6% |
| **NRXN1** | 924 | 132 | 218.4 | 496.0 | 181.2 | 396.8 | 3.09E-24 | 2.42E-21 | 12.5% |
| **RP1L1** | 924 | 132 | 221.8 | 472.6 | 193.2 | 371.8 | 5.14E-24 | 3.60E-21 | 12.5% |
| **NEB** | 924 | 132 | 224.5 | 453.7 | 197.0 | 370.3 | 4.03E-20 | 1.41E-17 | 12.5% |
| **FBN2** | 925 | 131 | 219.1 | 493.2 | 185.3 | 387.2 | 7.12E-24 | 4.62E-21 | 12.4% |
| **HCN1** | 925 | 131 | 229.4 | 420.9 | 206.7 | 352.4 | 1.36E-15 | 1.99E-13 | 12.4% |
| **LAMA2** | 927 | 129 | 230.2 | 417.5 | 215.2 | 318.7 | 4.85E-16 | 7.80E-14 | 12.2% |
| **PEG3** | 928 | 128 | 229.0 | 427.9 | 213.4 | 322.3 | 4.20E-19 | 1.21E-16 | 12.1% |
| **SSPO** | 928 | 128 | 225.2 | 455.5 | 196.3 | 378.1 | 1.72E-16 | 3.16E-14 | 12.1% |
| **DNAH7** | 928 | 128 | 236.0 | 377.6 | 227.5 | 275.3 | 5.72E-14 | 6.26E-12 | 12.1% |
| **DNAH11** | 929 | 127 | 219.7 | 497.3 | 183.9 | 397.0 | 3.19E-24 | 2.42E-21 | 12.0% |
| **OBSCN** | 929 | 127 | 228.6 | 432.6 | 206.4 | 352.5 | 1.59E-19 | 4.83E-17 | 12.0% |
| **CNTNAP2** | 929 | 127 | 228.1 | 436.2 | 200.5 | 374.8 | 4.78E-16 | 7.75E-14 | 12.0% |
| **MYH2** | 930 | 126 | 234.5 | 390.8 | 210.9 | 355.7 | 2.80E-11 | 1.56E-09 | 11.9% |
| **SORCS1** | 931 | 125 | 220.6 | 495.0 | 186.4 | 394.0 | 4.86E-22 | 2.27E-19 | 11.8% |
| **TNN** | 931 | 125 | 234.5 | 391.8 | 219.7 | 314.3 | 1.81E-12 | 1.47E-10 | 11.8% |
| **HERC2** | 932 | 124 | 225.3 | 462.4 | 193.2 | 392.6 | 2.01E-18 | 5.21E-16 | 11.7% |
| **PTPRD** | 932 | 124 | 225.0 | 464.5 | 193.3 | 390.8 | 4.49E-18 | 1.10E-15 | 11.7% |
| **CPS1** | 933 | 123 | 226.3 | 456.7 | 199.9 | 371.3 | 2.69E-21 | 1.14E-18 | 11.6% |
| **AHNAK** | 934 | 122 | 229.9 | 431.2 | 202.7 | 376.2 | 1.02E-14 | 1.28E-12 | 11.6% |
| **ADGRV1** | 934 | 122 | 227.6 | 448.2 | 194.3 | 400.2 | 4.55E-13 | 4.09E-11 | 11.6% |
| **ZNF804B** | 934 | 122 | 233.8 | 401.1 | 212.1 | 350.5 | 3.62E-11 | 1.95E-09 | 11.6% |
| **SPHKAP** | 935 | 121 | 228.5 | 443.7 | 201.4 | 376.1 | 2.52E-17 | 5.45E-15 | 11.5% |
| **NPAP1** | 935 | 121 | 228.5 | 443.0 | 200.4 | 380.8 | 2.29E-16 | 4.01E-14 | 11.5% |
| **MYH1** | 935 | 121 | 227.3 | 452.9 | 194.1 | 399.9 | 2.63E-15 | 3.67E-13 | 11.5% |
| **TENM1** | 935 | 121 | 232.6 | 411.7 | 207.9 | 365.7 | 1.27E-14 | 1.54E-12 | 11.5% |
| **ASTN1** | 936 | 120 | 224.7 | 474.5 | 199.8 | 365.3 | 2.80E-20 | 1.02E-17 | 11.4% |
| **COL6A3** | 937 | 119 | 224.6 | 477.7 | 187.7 | 412.7 | 3.29E-17 | 6.86E-15 | 11.3% |
| **NALCN** | 938 | 118 | 231.3 | 426.3 | 212.7 | 339.2 | 1.26E-14 | 1.54E-12 | 11.2% |
| **CTNNA2** | 940 | 116 | 232.1 | 423.7 | 211.1 | 351.6 | 1.72E-15 | 2.44E-13 | 11.0% |
| **ANKRD30A** | 940 | 116 | 233.0 | 416.3 | 208.4 | 368.2 | 1.82E-11 | 1.07E-09 | 11.0% |
| **VCAN** | 941 | 115 | 226.0 | 474.7 | 197.7 | 383.5 | 1.15E-16 | 2.22E-14 | 10.9% |
| **COL12A1** | 941 | 115 | 233.1 | 417.3 | 214.2 | 340.8 | 4.03E-13 | 3.65E-11 | 10.9% |
| **SYNE2** | 942 | 114 | 224.7 | 487.8 | 192.5 | 398.4 | 1.21E-16 | 2.31E-14 | 10.8% |
| **ADAMTS20** | 942 | 114 | 234.0 | 410.7 | 216.6 | 332.9 | 1.09E-14 | 1.35E-12 | 10.8% |
| **TPTE** | 943 | 113 | 235.6 | 399.5 | 220.0 | 320.9 | 2.08E-15 | 2.93E-13 | 10.7% |
| **ASPM** | 943 | 113 | 232.5 | 425.5 | 209.4 | 363.1 | 3.73E-13 | 3.39E-11 | 10.7% |
| **NF1** | 943 | 113 | 239.5 | 366.5 | 227.4 | 291.3 | 1.68E-07 | 3.03E-06 | 10.7% |
| **FMN2** | 944 | 112 | 235.4 | 402.5 | 215.5 | 345.5 | 1.04E-11 | 6.64E-10 | 10.6% |
| **MXRA5** | 945 | 111 | 228.6 | 462.1 | 206.6 | 358.3 | 2.15E-16 | 3.88E-14 | 10.5% |
| **ADGRL3** | 945 | 111 | 235.3 | 404.9 | 222.8 | 303.7 | 3.01E-16 | 5.07E-14 | 10.5% |
| **MYH8** | 945 | 111 | 231.2 | 440.1 | 212.7 | 341.0 | 5.37E-16 | 8.42E-14 | 10.5% |
| **MROH2B** | 945 | 111 | 229.2 | 457.0 | 200.7 | 389.1 | 1.27E-15 | 1.90E-13 | 10.5% |
| **PCDH10** | 945 | 111 | 230.2 | 448.2 | 211.4 | 342.9 | 5.41E-15 | 7.28E-13 | 10.5% |
| **PRDM9** | 945 | 111 | 234.5 | 411.8 | 222.5 | 301.4 | 1.11E-13 | 1.14E-11 | 10.5% |
| **TG** | 946 | 110 | 226.9 | 478.8 | 209.1 | 335.7 | 5.30E-20 | 1.72E-17 | 10.4% |
| **CDH9** | 946 | 110 | 234.0 | 417.4 | 220.1 | 314.2 | 1.12E-13 | 1.15E-11 | 10.4% |
| **CRB1** | 946 | 110 | 233.1 | 425.1 | 206.4 | 382.5 | 1.77E-11 | 1.05E-09 | 10.4% |
| **FLG2** | 947 | 109 | 228.3 | 468.9 | 206.3 | 359.1 | 4.14E-20 | 1.42E-17 | 10.3% |
| **PLXNA4** | 947 | 109 | 237.8 | 386.2 | 223.3 | 312.4 | 1.19E-10 | 5.42E-09 | 10.3% |
| **EPHA5** | 948 | 108 | 228.1 | 472.5 | 206.0 | 359.8 | 1.25E-21 | 5.68E-19 | 10.2% |
| **HYDIN** | 948 | 108 | 226.9 | 483.0 | 190.4 | 423.1 | 3.93E-15 | 5.37E-13 | 10.2% |
| **MUC5B** | 948 | 108 | 235.5 | 408.1 | 219.2 | 327.0 | 5.02E-14 | 5.53E-12 | 10.2% |
| **CDH12** | 949 | 107 | 228.9 | 468.4 | 203.9 | 374.9 | 2.35E-17 | 5.15E-15 | 10.1% |
| **MRC1** | 950 | 106 | 228.2 | 476.6 | 195.6 | 410.0 | 9.79E-18 | 2.28E-15 | 10.0% |
| **ASXL3** | 950 | 106 | 232.4 | 438.8 | 216.6 | 327.5 | 4.09E-16 | 6.82E-14 | 10.0% |
| **DNAH3** | 950 | 106 | 228.9 | 470.4 | 198.4 | 401.6 | 6.49E-14 | 6.93E-12 | 10.0% |
| **UNC13C** | 950 | 106 | 233.4 | 430.3 | 210.8 | 364.9 | 2.28E-12 | 1.76E-10 | 10.0% |
| **KIAA1109** | 950 | 106 | 234.7 | 418.6 | 213.5 | 356.5 | 1.80E-11 | 1.06E-09 | 10.0% |

n1, n2, mean1, mean2 and sd1, sd2 represent the number, mean WES-TMB and standard division of WES-TMB based on the 1,026 NSCLC patients in the groups with mutated gene and their wild-type counterparts. The mean WES-TMB were tested with Wilcoxon test (p value) adjusted by the Bonferroni method (p_Adjust). The mutation frequencies of these genes in NSCLC were calculated as Mutation Freq.

**Table S4. 23 genes and the corresponding CDS length.**

| **Gene Symbol** | **CDS length (nucleotides)** |
| --- | --- |
| UNC13C | 6645 |
| HMCN1 | 16908 |
| ZNF536 | 3903 |
| KMT2D | 16614 |
| USH2A | 15609 |
| XIRP2 | 11355 |
| PCDH15 | 5034 |
| AHNAK2 | 17388 |
| ADGRL3 | 4410 |
| RELN | 10377 |
| NF1 | 8520 |
| TTN | 80781 |
| ADGRG4 | 9243 |
| CUBN | 10872 |
| CACNA1E | 6942 |
| MRC1 | 4371 |
| COL11A1 | 5304 |
| NAV3 | 7158 |
| CSMD1 | 10695 |
| APOB | 13692 |
| CSMD3 | 10617 |
| COL22A1 | 4881 |
| EPHA5 | 3048 |

**2. Supplementary Figures**

**
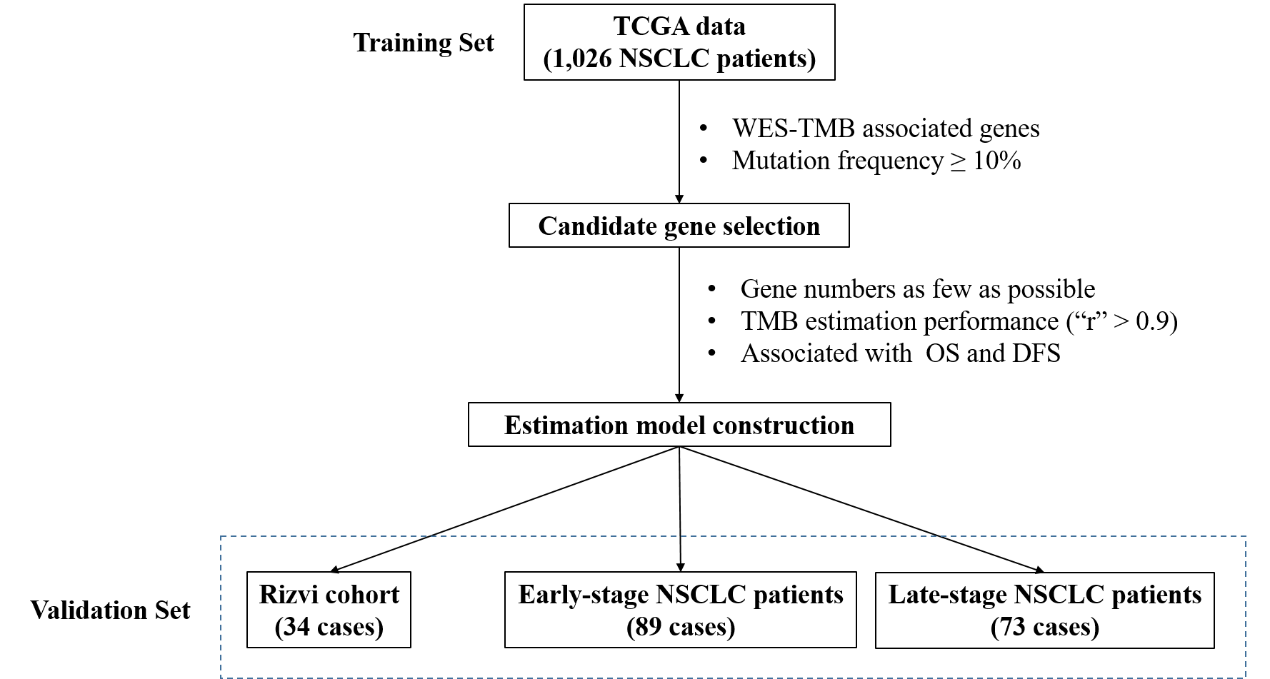
Figure S1.**

**Fig. S1** Flowchart of the construction of estimation model.

**
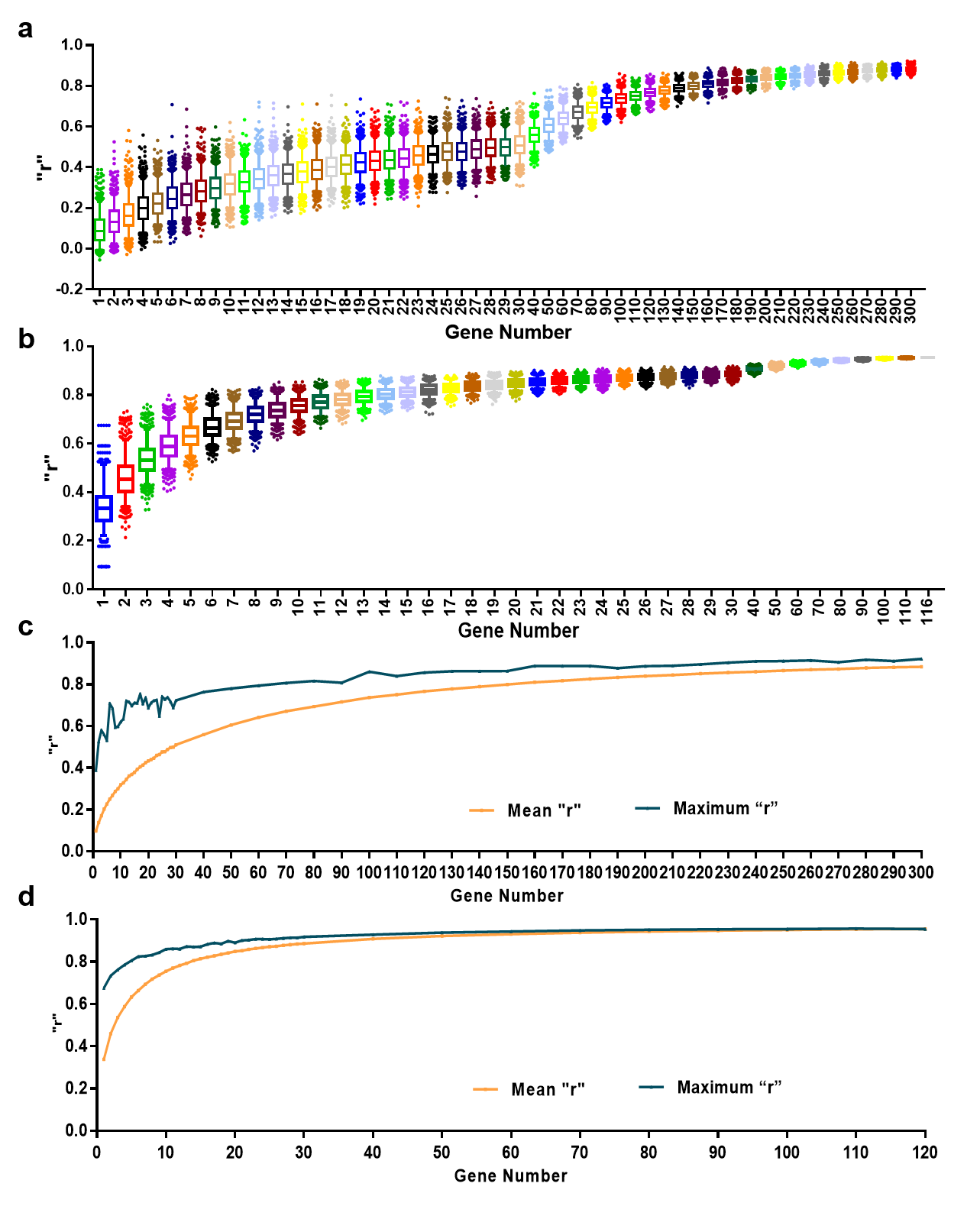
Figure S2**

**Fig. S2** The correlation of WES-TMB and TMB, as estimated by different gene panels. **a, b** Correlation is represented by the Pearson correlation coefficient (“r”). Genes used for the mutation model construction were either from unselected genes (a) or selected 116 genes (b). **c, d** The maximum and mean Pearson correlation coefficient by using unselected one to 300 genes (c) or selected one to 116 (d).

**Figure**
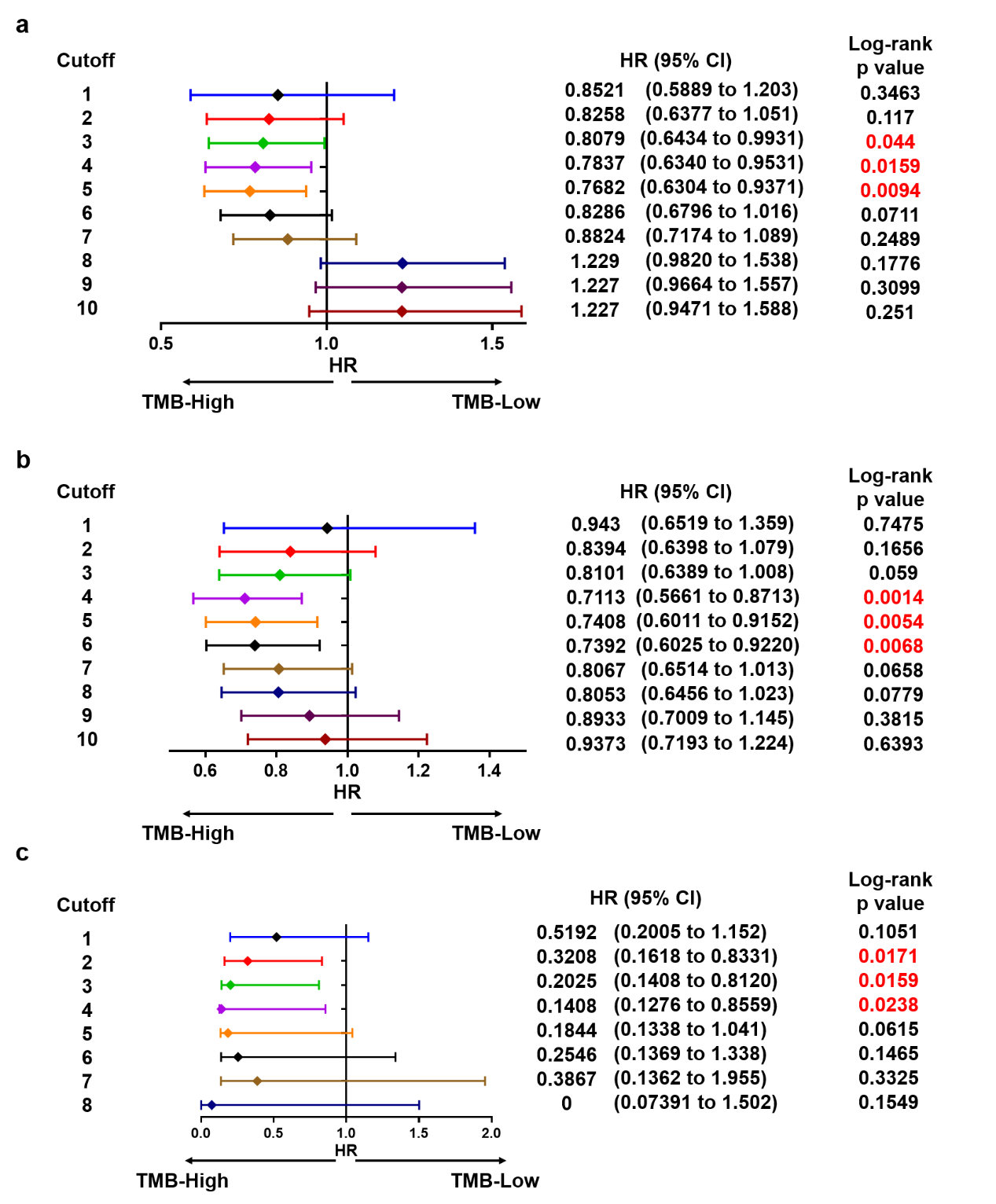
**S3**

**Fig. S3** Forest plots of HRs for OS and DFS in the TCGA and 89 early-stage NSCLC patients study cohort. **a, b** The association of 23 genes with OS (a) DFS (b) at different cut-points in TCGA cohort. **c** The association of the 23 genes with DFS of the 89 early-stage NSCLC patients.

**
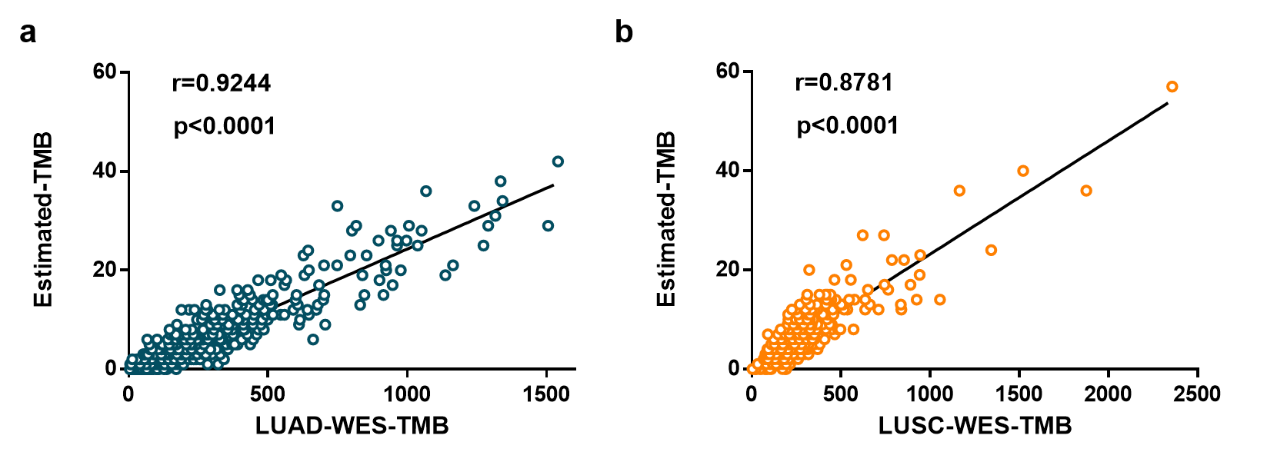
Figure S4**

**Fig. S4** The performance of 23-gene based TMB estimation model for the LUAD and LUSC subtypes of NSCLC (TCGA data). **a, b** The correlations of estimated TMB with WES-TMB of LUAD (LUAD-WES-TMB) and LUSC (LUSC-WES-TMB).

**
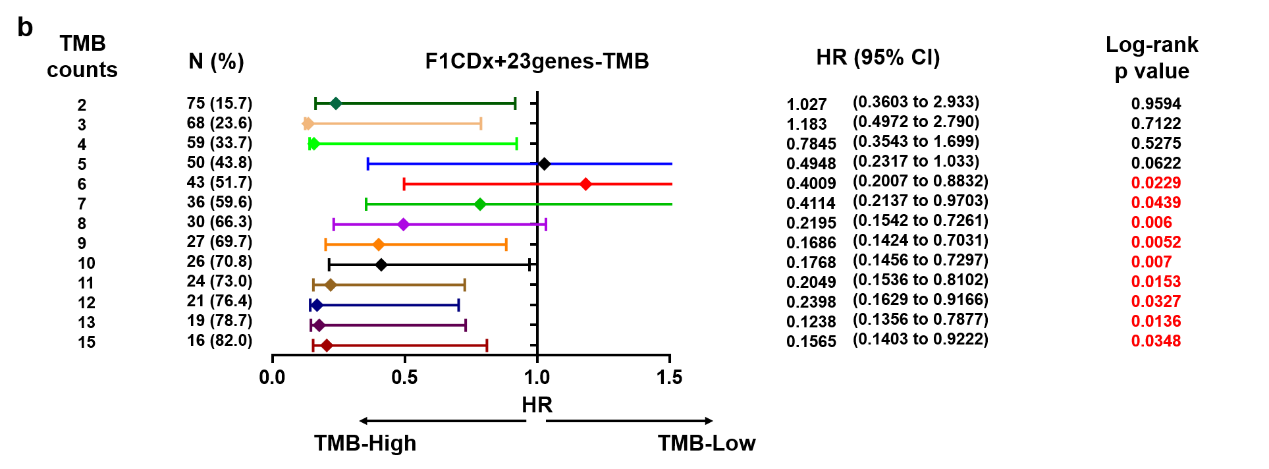

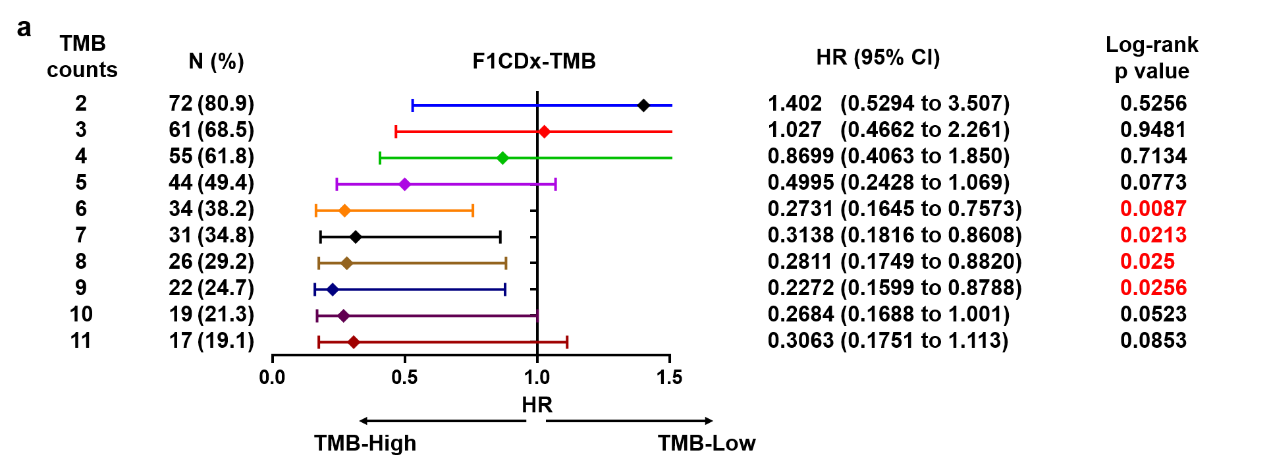

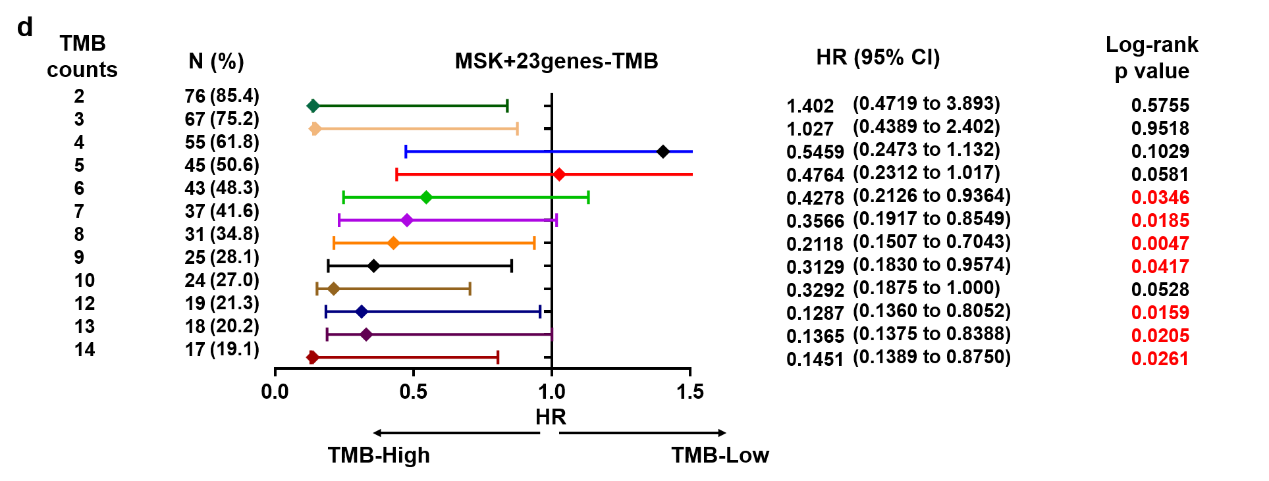

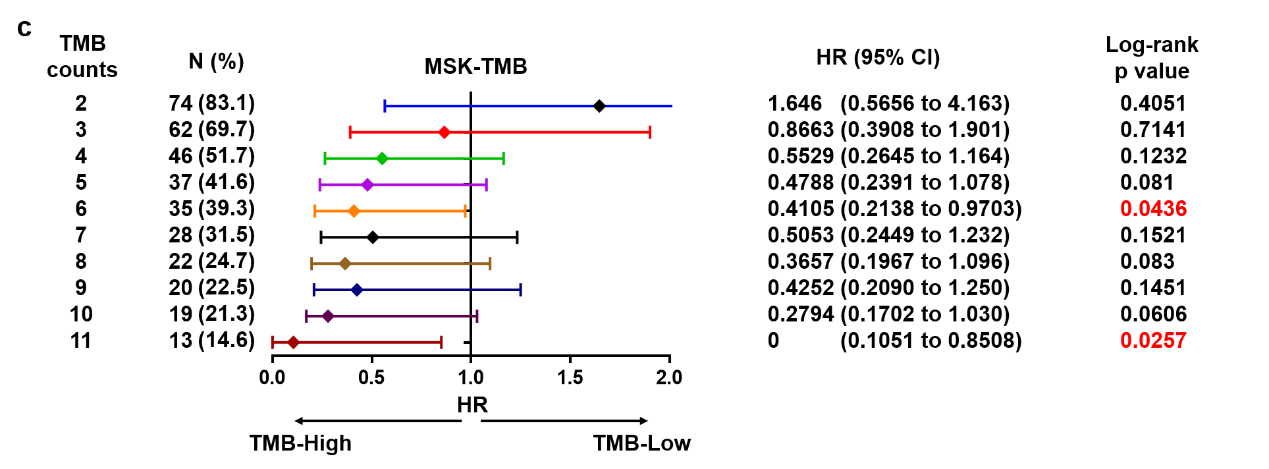
Figure S5**

**Fig. S5** Forest plots of HRs for DFS in the 89 early-stage NSCLC patients study cohort. **a, c** The association of F1CDx-405 genes (a) and MSK 414 genes (c) with DFS at different cut-points. **b, d** The association of the incorporated panels of 23 genes with F1CDx-405 genes (b) and MSK 414 genes (d) with DFS. Red indicated log-rank p value <0.05.


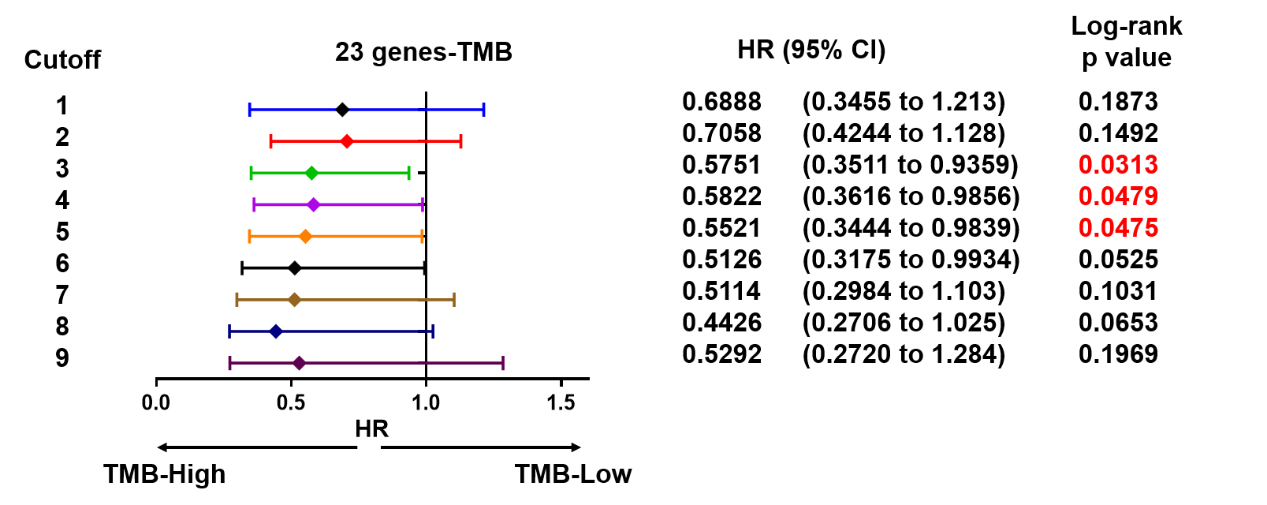
**Figure S6**

**Fig. S6** Forest plots of HRs for PFS of the 73 NSCLC patients in ZS immunotherapy cohort.

**
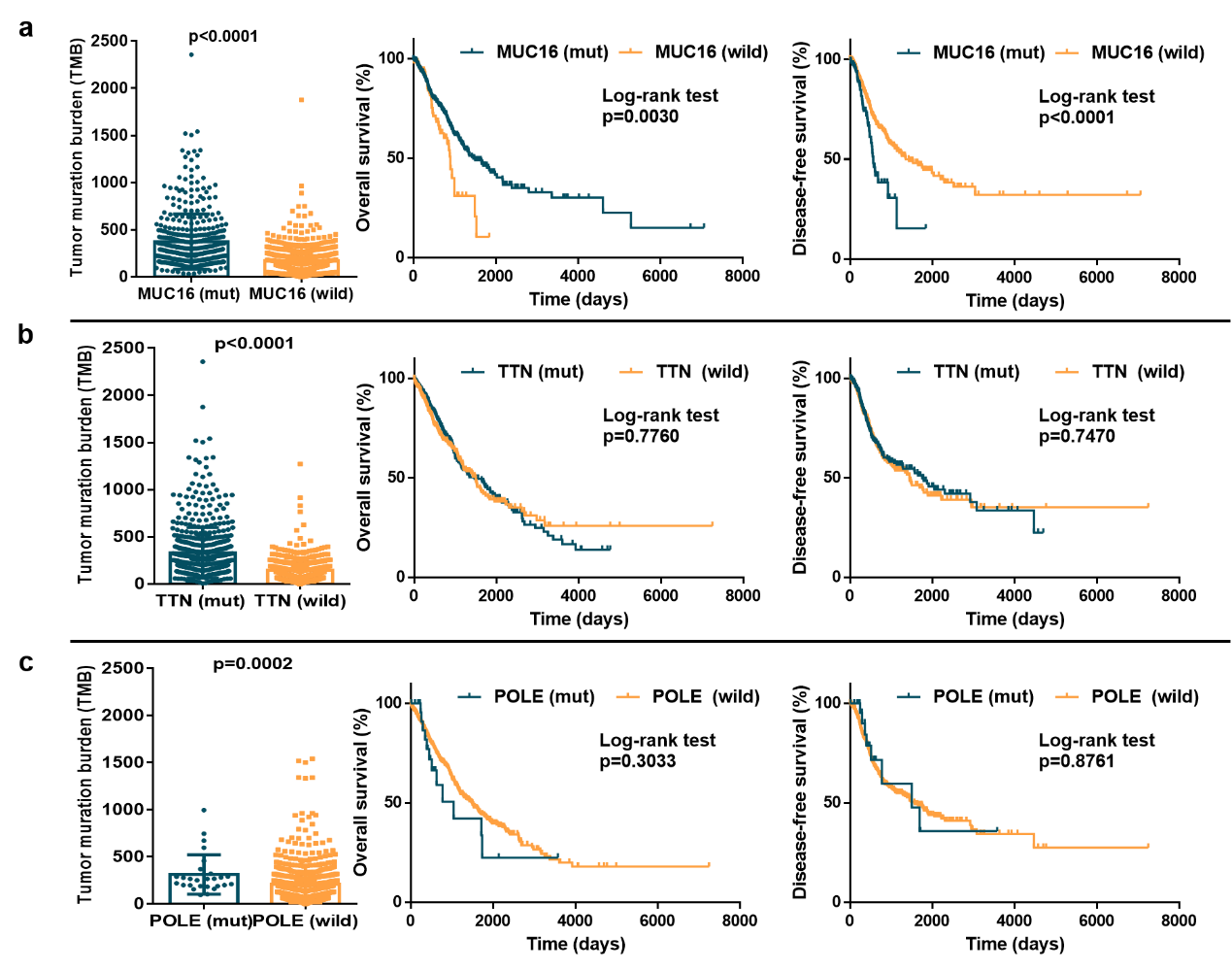
Figure S7**

**Fig. S7** WES-TMB is shown based on MUC16 (a), TTN (b) and POLE (c) mutation status. The **s**tatistics are based on a 2-tailed Mann-Whitney U test. The box plots show the median value with the min to max range. Survival analysis is performed by using Kaplan–Meier curves, with a p-value determined by a log-rank test.

**
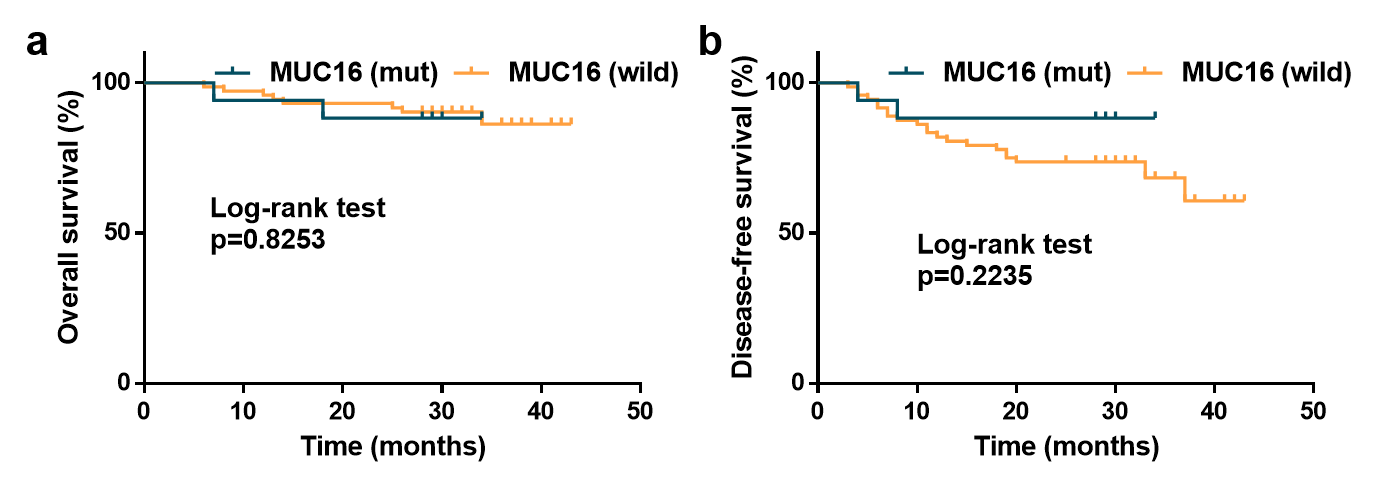
Figure S8**

**Fig. S8** The correlation of MUC16 mutation status with overall survival (a) and disease-free survival (b) based on the 89 early-stage NSCLC patients. The log-rank test p-values are 0.8253 and 0.2235 for OS and DFS, respectively.

**
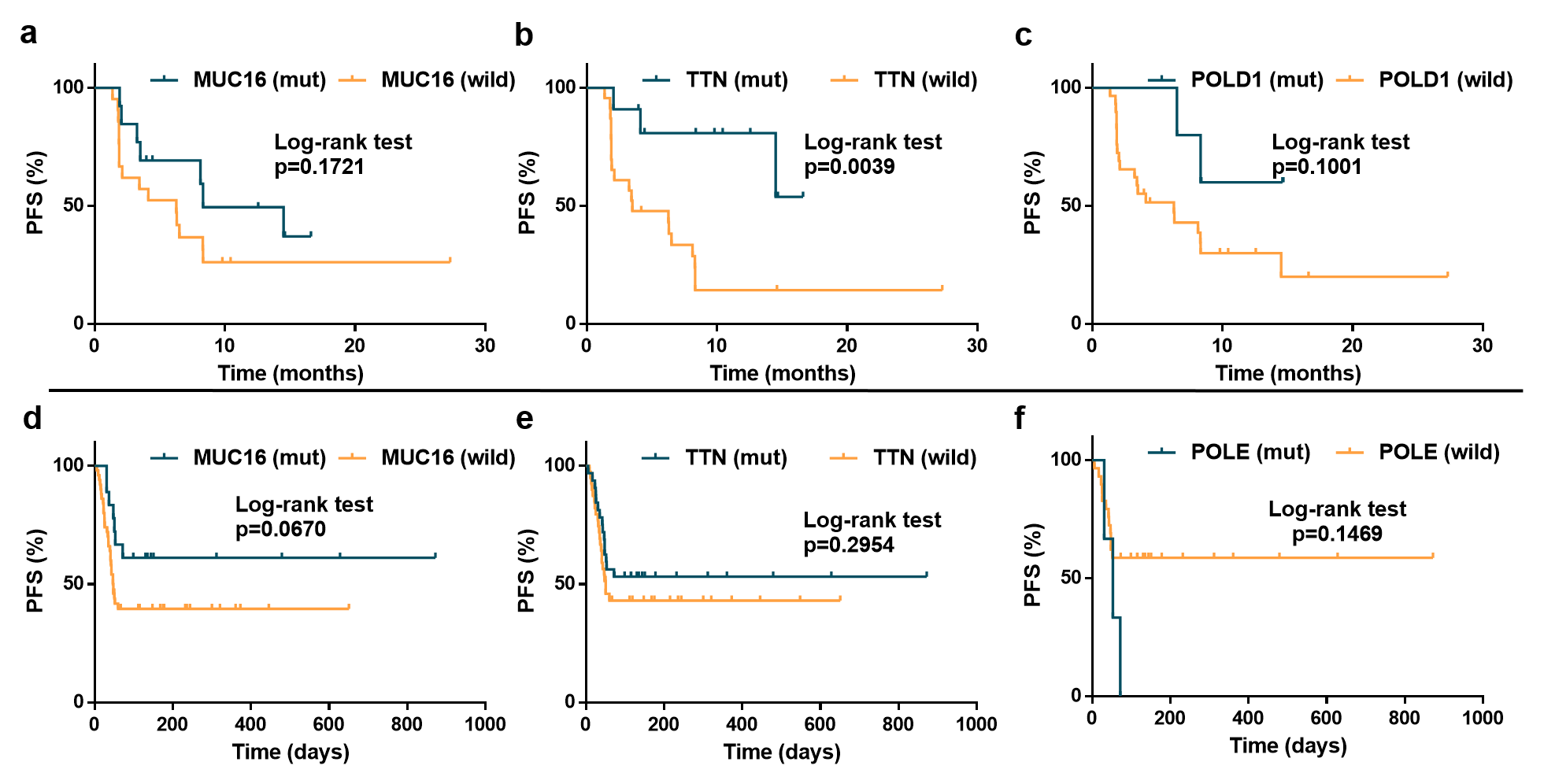
Figure S9**

**Fig. S9** The correlation of MUC16, TTN and POLD1 mutation status with progression-free survival (PFS) based on the *Rizvi* cohort and our immunotherapy cohort. **a-c** Analysis of the *Rizvi* cohort. **d-f** Analysis of our immunotherapy patients.

**
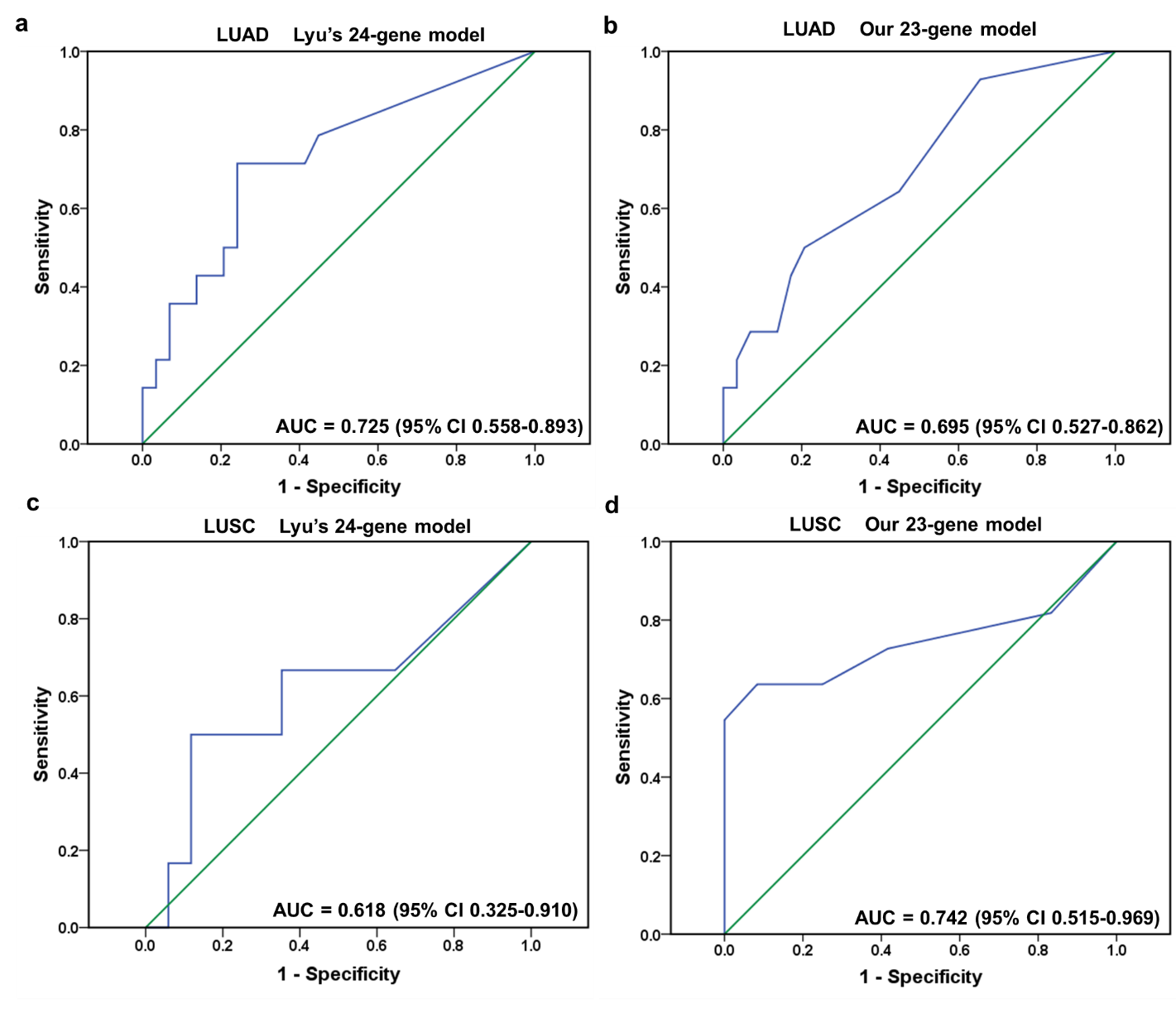
Figure S10**

**Fig. S10** Comparison of predictive performance of response to ICIs by our 23-gene panel with Lyu’s 24-gene panel. **a, b** Plots of ROC results for distinguishing LUAD patients (n=43) who received DCB and NDB in the ZS immunotherapy cohort by Lyu’s 24-gene panel (a) and our 23-gene panel (b). **c, d** Plots of ROC results for distinguishing LUSC patients (n=12) who received DCB and NDB in the ZS immunotherapy cohort by Lyu’s 24-gene panel (c) and our 23-gene panel (d). The ROC curves were created by plotting the sensitivity (i.e., true positive rate) against 1-specificity (i.e., false positive rate). The blue line in each plot represents the area under the curve (AUC).
